# A Pair-Electrosynthesis for Formate at Ultra-Low Voltage Via Coupling of CO_2_ Reduction and Formaldehyde Oxidation

**DOI:** 10.1007/s40820-022-00953-y

**Published:** 2022-11-01

**Authors:** Mengyu Li, Tehua Wang, Weixing Zhao, Shuangyin Wang, Yuqin Zou

**Affiliations:** 1 State Key Laboratory of Chemo/Bio-Sensing and Chemometrics, College of Chemistry and Chemical Engineering, Advanced Catalytic Engineering Research Center of the Ministry of Education, Hunan University, Changsha, 410082 People’s Republic of China; 2 School of Chemistry and Chemical Engineering, Jishou University, Jishou, 416000 Hunan People’s Republic of China

**Keywords:** Formate pair-electrolysis, Electrochemical CO_2_ reduction, Formaldehyde oxidation reaction, Membrane electrode assembly, Flow cell

## Abstract

Formate can be synthesized electrochemically by CO_2_ reduction reaction (CO_2_RR) or formaldehyde oxidation reaction (FOR). The CO_2_RR approach suffers from kinetic-sluggish oxygen evolution reaction at the anode. To this end, an electrochemical system combining cathodic CO_2_RR with anodic FOR was developed, which enables the formate electrosynthesis at ultra-low voltage. Cathodic CO_2_RR employing the BiOCl electrode in H-cell exhibited formate Faradaic efficiency (FE) higher than 90% within a wide potential range from − 0.48 to − 1.32 *V*_RHE_. In flow cell, the current density of 100 mA cm^−2^ was achieved at − 0.67 *V*_RHE_. The anodic FOR using the Cu_2_O electrode displayed a low onset potential of − 0.13 *V*_RHE_ and nearly 100% formate and H_2_ selectivity from 0.05 to 0.35 *V*_RHE_. The CO_2_RR and FOR were constructed in a flow cell through membrane electrode assembly for the electrosynthesis of formate, where the CO_2_RR//FOR delivered an enhanced current density of 100 mA cm^−2^ at 0.86 V. This work provides a promising pair-electrosynthesis of value-added chemicals with high FE and low energy consumption.

**Supplementary Information:**

The online version contains supplementary material available at 10.1007/s40820-022-00953-y.

## Highlights

- Pair electrosynthesis of formate was achieved by combining CO_2_ reduction reaction (CO_2_RR) and formaldehyde oxidation reaction (FOR) in the membrane electrode assembly.
- Replacing oxygen evolution reaction with thermodynamically more favorable FOR reduces energy consumption.
- CO_2_RR//FOR exhibits excellent electrochemical performance (100 mA cm^−2^ @ 0.86 V), electricity consumption normalized by the mass of formate was 0.413 Wh g^−1^.

**Supplementary Information:**

The online version contains supplementary material available at 10.1007/s40820-022-00953-y.

## Graphical abstract

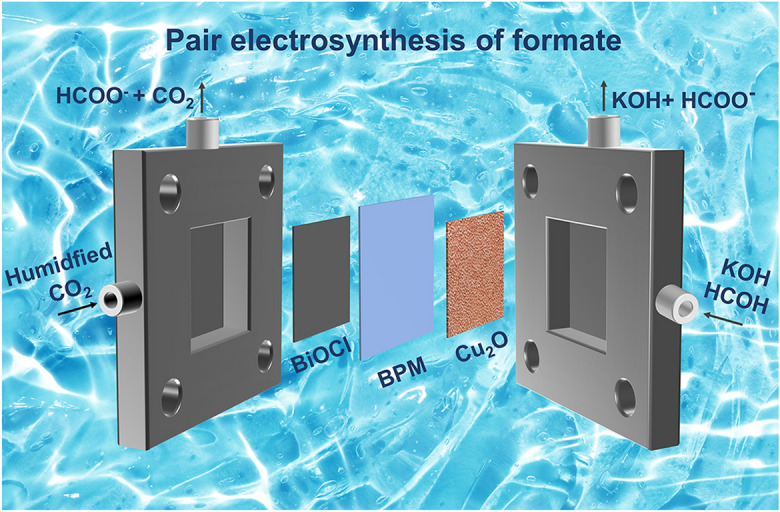

## Introduction

The excessive exploitation and utilization of fossil fuels trigger serious environmental issues and energy crises which have aroused widespread concerns [1]. Electrochemical CO_2_ reduction reaction (CO_2_RR), powered by intermittent renewable energy resources (such as solar, wind, or tide energy) meanwhile producing valuable liquid fuels or chemicals, forms a carbon–neutral cycle [2–6]. A recent technical and economic analysis of CO_2_RR revealed that the high market competitiveness of formate as the most economically viable product with the advantages of non-toxicity, safety, and non-flammability, which can be used directly in formic acid fuel cells or as hydrogen storage carrier [7–12]. The market demand for formate has increased steadily since 2013 (697,000 tons per year), which is estimated to reach approximately 1 million tons in 2030 [13]. Therefore, it is of great significance to develop efficient electrocatalysts for CO_2_ to formate. Bismuth-based catalyst has the advantage of safety, low cost, environmental friendliness, and facile synthesis, achieving high selectivity for CO_2_ to formate. Enhancing the p-orbital delocalization of metallic Bi obtained by electrochemical reduction in BiOCl boosts CO_2_RR to formate [14]. Sargent et al*.* [15] demonstrated that BiOBr-templated catalyst preferentially exposed high CO_2_RR activity Bi (110) facets. A lattice disordering defect-rich Bi derived from Bi_2_S_3_ was reported to have a high Faradaic efficiency (FE) of CO_2_RR to formate [16]. The bismuth-based catalyst reduces to metallic bismuth under reduction potential; structural transformation of the bismuth-based catalyst generates surface vacancies and atomic-scale disordering, which is favorable for CO_2_RR to formate [13, 15, 16].

Cathodic CO_2_RR is usually coupled with oxygen evolution reaction (OER) occurring at the anode. OER is a four-electron transfer uphill reaction, suffering from sluggish reaction kinetics thereby requiring a high overpotential [17]. It is worth noting that thermodynamic analysis demonstrated that OER consumes 94.5% of the electricity energy in the CO_2_RR conversion system [18]. This adds the urgency to develop thermodynamically favorable reactions for high-efficient energy conversion in the production of value-added chemicals. In recent years, electrooxidation of organic molecules, such as urea [19], methanol [18, 20], benzyl alcohol [21], glycerol [22], and 5-hydroxymethylfurfural (HMF) [23, 24], has been regarded as a promising alternative to OER in terms of low cell voltage and energy consumption in the CO_2_RR conversion system. Up to date, the existing coupling systems mostly deliver relatively high cell voltage (usually higher than 2 V) [19, 21, 23]. Meanwhile, infiltration and separation of the electrode products have also been major problems in those coupling systems. These issues can be well-solved by paired electrosynthesis.

Electrosynthesis of formate was achieved by combining CO_2_RR with methanol oxidation reaction (MOR) [18, 20]. Ni-based metal organic framework (MOF) nanosheet arrays for MOR and Bi-MOF derived Bi-enes for CO_2_RR, requiring a high cell voltage of 2.13 V to deliver 10 mA cm^−2^ [20]. However, the relatively low current density under 50 mA cm^−2^ limits the practical application for formate electrosynthesis. In our previous work, the oxidation of the aldehyde to acid accompanied by the formation of H_2_ was successfully achieved in alkaline electrolytes at an ultra-low potential catalyzed by a Cu/Cu foam catalyst [25, 26]. Cu foam is a three-dimensional highly porous substrate that facilitates mass transfer and gas desorption. Plasma oxidation followed by in situ electroreduction is a facile and efficient strategy to improve the roughness of Cu foam substrate and increase the number of active sites. Electrosynthesis of formate can also be realized by coupling CO_2_RR and glycerol oxidation reaction (GOR). Compared with CO_2_RR//GOR, CO_2_RR//FOR has plenty of advantages. Firstly, the thermodynamic equilibrium potential of FOR to formate and H_2_ is only − 0.224 *V*_RHE_ (vs. reversible hydrogen electrode), much lower than OER (1.23 *V*_RHE_) and GOR to formate (0.14 *V*_RHE_). Secondly, the oxidation of formaldehyde to formate and H_2_ is a single electron transfer reaction, and the oxidation of glycerol to formate is an eight-electron transfer reaction. Compared with CO_2_RR//GOR, CO_2_RR-combined FOR is accompanied by the formation of H_2_, requires lower cell voltage to reach the same current density produces more formate when passing the same amount of charge, and possesses more significant profit. Thus, the FOR provides a promising route to convert formaldehyde into highly valuable formate and H_2_, but it can also reduce the overall energy consumption of CO_2_RR conversion devices by replacing the anodic OER.

Therefore, in this work, paired electrosynthesis was carried out by combining the cathodic CO_2_RR and the anodic FOR to the transformation of formaldehyde into formate at low cell voltage with high energy conversion. The cathodic CO_2_RR and anodic FOR were investigated independently on the BiOCl nanosheet and Cu_2_O electrode, respectively. Afterward, CO_2_RR and FOR were combined and constructed in the membrane electrode assembly (MEA) to improve the cell performance for formate electrosynthesis. With the aid of MEA, the cell voltage only needs 0.86 V to reach 100 mA cm^−2^. The as-designed CO_2_RR//FOR electrolyzer is expected to reduce energy consumption, produce value-added chemicals, and promote a sewage-treatment system toward a carbon–neutral goal.

## Experimental Section

### Syntheses of BiOCl

BiOCl nanosheet was prepared by hydrothermal method [27]. Briefly, 5 mmol bismuth nitrate pentahydrate and 5 mmol potassium chloride were dissolved in a mixed solution consisting of 70-mL deionized water and 5-mL ethylene glycol under continuous stirring for 30 min. After that the above solution was transferred into a 100-mL autoclave and heated at 160 °C for 24 h. After naturally cooling to room temperature, the obtained product was collected by filtering, washed with deionized water, and finally drying in vacuum at 60 °C.

### Syntheses of Cu1 (CuO/Cu_2_O) and Cu2 (Cu_2_O)

Cu foam was first treated with oxygen plasma and then in situ electrochemical reduction. Briefly, a piece of Cu foam was placed in the middle of quartz tube, after that reactor was filled with O_2_ at a flow rate of 10 sccm. Cu foam was treated with O_2_ plasma with the power of 300 W and gas pressure of 15 Pa, the front and the back were treated for 5 min each, as-obtained catalyst is recorded as Cu1 (CuO/Cu_2_O). Before FOR, in situ electrochemical reduction of Cu1 was performed at − 0.4 *V*_RHE_ in 1 M KOH electrolyte for 400 s, as-obtained catalyst is recorded as Cu2 (Cu_2_O).

### Physical Characterization

Morphology of as-prepared catalysts was characterized by scanning electron microscopy (SEM, Hitachi, S4800) and transmission electron microscopy (TEM, FEI, Tecnai G2 F20). Crystal structure was examined by X-ray diffraction (XRD) on a Bruker D8-Advance X-ray diffractometer with a Cu K*α* source (1.54056 Å). X-ray photoelectron spectroscopy (XPS) measurement was recorded on Escalab 250Xi photoelectron spectrometer.

### Electrochemical Measurement

All the electrochemical measurements were performed on CHI760D or Ivium electrochemical workstation at room temperature. CO_2_RR and FOR were carried out in an H-cell, and proton exchange membrane (PEM, Nafion 117) acts as separator. For CO_2_RR, each compartment contained 35 mL 0.5 M KHCO_3_ electrolyte. Working electrode was BiOCl or commercial Bi power loaded on carbon paper (1 cm^2^), Pt mesh (1 cm^2^), and Ag/AgCl (saturated KCl solution) which were used as counter electrode and reference electrode, respectively. All the applied potentials were converted to RHE according to Nernst equation, *E*(RHE) = *E*(Ag/AgCl) + 0.197 V + 0.0591 V × pH. Typically, 5 mg catalyst and 1 mg Ketjenblack carbon were dispersed on 0.95-mL ethanol and 50-μL Nafion solution (5 wt%) by sonicating for 30 min, and 200 μL of homogeneous ink was drop-cast onto 1 cm^2^ carbon paper. Before electrochemical test, electrolyte was bubbled with CO_2_ for at least 30 min under magnetic stirring (400 rpm), and flow rate of CO_2_ was 20 sccm. Linear sweep voltammetry (LSV) was measured at the scan rate of 10 mV s^−1^ in Ar (pH = 8.66)- or CO_2_ (pH = 7.45)-saturated 0.5 M KHCO_3_, polarization curves without *iR*-correction. Electrochemical impedance spectroscopy (EIS) measured at the potential of − 0.76 *V*_RHE_ with frequencies ranging from 0.01 Hz to 100 kHz, and the amplitude was 5 mV. Electrochemical surface area (ECSA) can be calculated by cyclic voltammetry (CVs). CV was performed at potentials ranging from 0.4 to 0.5 V versus Ag/AgCl at the scan rate of 20, 40, 60, 80, and 100 mV s^−1^.

1 $${\text{ECSA}} = R_{{\text{f}}} \times S = \frac{{C_{{{\text{dI}}}} }}{{C_{{\text{S}}} }} \times S$$

where *S* is the geometric area of working electrode (1 cm^2^), *R*_f_ is the roughness factor of working electrode, and *C*_dI_ is double-layer capacitance.

2 $${\Delta }j = 0.5 \times \left( {j_{{\text{a}}} - j_{{\text{c}}} } \right)$$

where *j*_a_ and *j*_c_ is the current density of anode and cathode, respectively. By plotting Δ*j* at 0.45 V versus Ag/AgCl against scan rate, slope is *C*_dI_.

For FOR, each compartment contained 35 mL 1 M KOH, and the anode with the addition of 0.1 M formaldehyde. Working electrode was Cu2 (Cu_2_O, 1 cm^2^), and Pt mesh (1 cm^2^) and saturated calomel electrode (SCE) were used as counter electrode and reference electrode, respectively. All the applied potentials were converted to the RHE, *E*(RHE) = *E*(SCE) + 0.241 V + 0.0591 V × pH. LSV performed at the scan rate of 5 mV s^−1^, polarization curves without *iR*-compensation. EIS measured at potentials ranging from − 0.25 to − 0.05 *V*_RHE_.

CO_2_RR//OER and CO_2_RR//FOR full cells were performed in an H-cell, CO_2_-saturated 0.5 M KHCO_3_ was used as CO_2_RR electrolyte, 1 M KOH as OER electrolyte, and 1 M KOH with the addition of 0.1 M formaldehyde as FOR electrolyte. Nafion 117 separates cathode and anode. All electrochemical measurements in this part without *iR*-compensation.

### Fabrication of Flow Cell

For CO_2_RR, the custom-designed flow cell consisted of BiOCl-loaded gas diffusion electrode (GDE) as the cathode and IrO_2_ loaded on GDE as the anode. Typically, 4 mg BiOCl and 1 mg Ketjenblack carbon were dispersed on 0.95-mL ethanol and 50-μL Nafion solution (5 wt%) by sonicating for 30 min to form a homogeneous ink. Then, all of the above solutions were drop-casted onto GDE (YLS 30T, Toray) with an area of 4 cm^2^ (2 × 2 cm^2^ with the loading of 1 mg cm^−2^), obtaining a working electrode used for CO_2_RR tests. Cathode and anode were separated by anion exchange membrane (AEM), and Ag/AgCl electrode (saturated KCl solution) was placed inside the cathode compartment. CO_2_ flow rate was 20 sccm controlled by mass flow controller. 1 M KOH was used as catholyte and anolyte with the flow rate of 3.3 sccm, and electrolyte was circulated through flow cell using peristaltic pump.

CO_2_RR//FOR full cell was performed in a liquid-phase flow cell. Cu_2_O (1 × 1 cm^2^) was used as the anode, BiOCl loaded on GDE as the cathode, and AEM as the separator. 1 M KOH was used as CO_2_RR electrolyte with the flow rate of 3.3 sccm, and 1 M or 2 M KOH with the addition of 0.1 M formaldehyde acts as FOR electrolyte with the flow rate of 82.4 sccm. Catholyte and anolyte circulates in the groove of the plate through a peristaltic pump.

CO_2_RR//FOR full cell also performed in a gas-phase flow cell with the aid of MEA. The CO_2_ is pre-humidified before flow into the cathode plate to provide H^+^ and keep the ion exchange membrane moist. Cu_2_O was used as the anode, and BiOCl loaded on GDE was used as the cathode. 1 M KOH with the addition of 0.1 M formaldehyde as FOR electrolyte with the flow rate of 133.9 sccm, bipolar membrane (BPM) acts as solid electrolyte.

### Product Analysis

Liquid product of CO_2_RR to formate is quantitatively analyzed by high-performance liquid chromatography (HPLC, Shimadzu) using differential refractive index detector (RID) equipped with an organic acid column. The temperature of column is 60 °C, and the mobile phase is 5 mM H_2_SO_4_ with the flow rate of 0.4 mL min^−1^. Liquid product formate produced by FOR is quantitatively analyzed by nuclear magnetic resonance (NMR, Bruker 400 MHz) using internal standard method. Briefly, electrolyte after electrolysis was uniformly mixed with 0.5 M H_2_SO_4_ until the mixed electrolyte was neutral. 0.5-mL mixed electrolyte was uniformly mixed with 0.1-mL deuterated water (D_2_O) and 0.1-mL dimethyl sulfoxide (DMSO, 40 μL diluted to 100 mL by water). The one-dimensional ^1^H NMR spectroscopy uses pre-saturation method to suppress water peak. Liquid product methanol produced by formaldehyde disproportionation is quantitatively analyzed by gas chromatography (GC 2014, Shimadzu) equipped with flame ionization detector (FID), and the initial temperature of column is 40 °C.

Gas product of CO_2_RR and FOR is quantitatively analyzed by GC equipped with a thermal conductivity detector (TCD) and FID, and N_2_ was used as the carrier gas.

### Calculation of Faradaic Efficiency (FE)

FE of CO_2_RR and FOR liquid product is calculated as follows:

3 $${\text{FE}} = \frac{{Q_{{{\text{HCOOH}}}} }}{{Q_{{{\text{total}}}} }} \times 100\% = \frac{nZF}{{it}} \times 100\%$$

For CO_2_RR, *Z* = 2 is the number of transfer electrons to form 1 mol formate, *F* is Faraday constant (96,485 C mol^−1^), *n* is the moles of formate, *t* is electrolysis time (s), and *i* is the recorded current (*A*). For FOR, *Z* = 1 is the number of transfer electrons to form 1 mol formate.

FE of CO_2_RR and FOR gas product is calculated as follows:

4 $${\text{FE}} = \frac{Q}{{Q_{{{\text{total}}}} }} \times 100\% = \frac{{Z \times F \times V \left( {\frac{{{\text{mL}}}}{{{\text{min}}}}} \right) \times v \left( {{\text{vol}}\% } \right) \times P \left( {{\text{Pa}}} \right)}}{i \left( A \right) \times R \times T \left( K \right)}$$

For CO_2_RR, *Z* = 2 is the number of transfer electrons to form 1 mol H_2_. For FOR, *Z* = 2 is the number of transfer electrons to form 1 mol H_2_. *t* is electrolysis time (s), *V* is the gas flow rate (mL min^−1^), and *v* (vol%) is the volume concentration of CO or H_2_ in the outlet gas.

### Calculation of Production Rate (PR)

PR (mmol h^−1^ cm^−2^) of formate produced by CO_2_RR or FOR is calculated as follows:

5 $${\text{PR}} = \frac{n}{t \times S} \times 100\% = \frac{C \times V}{{t \times S}} \times 100\%$$

where *C* (mmol L^−1^) is the molar concentration of formate in the electrolyte, *V* (L) is the volume of electrolyte, and *t* (h) is electrolysis time and *S* (cm^2^) is geometric area of working electrode.

### Calculation of Single-Pass Conversion Efficiency (SOEC)

SOEC of CO_2_ to formate is calculated as follows:

6 $${\text{SPCE}} = \frac{{\frac{{Q \times {\text{FE}}}}{{{\text{ZF}}}} \times 22.4 \left( {L {\text{mol}}^{ - 1} } \right)}}{{V \left( {L \min^{ - 1} } \right) \times t \left( { \min } \right)}} = \frac{{i \times {\text{FE}} \times 60 \left( {s \min^{ - 1} } \right) }}{{{\text{ZF}}}} \times \frac{{22.4 \left( {L {\text{mol}}^{ - 1} } \right)}}{{V \left( {L \min^{ - 1} } \right)}}$$

where *Q* (C) is the total amount of charge passed through the system, *V* (L min^−1^) is the CO_2_ flow rate, *t* is the electrolysis time (min), *Z* = 2 is the number of transfer electrons to form 1 mol formate, and *i* is the record current (A).

#### Thermodynamic Analysis

Reaction standard redox potential can be calculated according to the following equation:

7 $${\Delta }G^{0} = - nE^{0} {\text{F}}$$

where Δ*G*^0^ (kJ mol^−1^) is the standard Gibbs free energy, *n* is the number of transfer electrons, *F* is Faraday constant, and *E*^θ^ is the standard redox potential. Reaction standard Gibbs free energy can be calculated according to Hess’s law (Table S1). Nernst equation is used to calculate *V*_RHE_:

8 $$E = E^{0} + 0.059 \times {\text{pH}}$$

9 $${\text{Cathode:}}\;{\text{CO}}_{2} + 2{\text{H}}^{ + } + 2{\text{e}}^{ - } \to {\text{HCOOH}}\quad \Delta G^{0} = 33.01\;{\text{kJ }}\;{\text{mol}}^{{ - 1}}$$

10 $$E^{0} = - 0.17 V_{{{\text{RHE}}}}$$

11 $${\text{Anode:}}\;2{\text{HCOH}} + 2{\text{OH}}^{ - } - 2{\text{e}}^{ - } \to 2{\text{HCOOH}} + {\text{H}}_{2} \quad \Delta G^{0} = - 203.152\;{\text{kJ}}\;{\text{ mol}}^{{ - 1}}$$

12 $$E^{0} = - 0.224 V_{{{\text{RHE}}}}$$

13 $${\text{Anode:}}\;2{\text{H}}_{2} {\text{O}} \to 4{\text{H}}^{ + } + 4{\text{e}}^{ - } + {\text{O}}_{2} \quad \Delta G^{0} = 237.129\;{\text{kJ}}\;{\text{ mol}}^{{ - 1}}$$

14 $$E^{0} = 1.23\; V_{{{\text{RHE}}}}$$

15 $${\text{Overall:}}\;2{\text{H}}_{2} {\text{O}} + {\text{CO}}_{2} + 2{\text{HCOH}} \to 3{\text{HCOOH}} + H_{2} \quad \Delta G^{0} = - 10.373\;{\text{kJ }}\;{\text{mol}}^{{ - 1}}$$

16 $$E^{0} = - 0.054\; V$$

17 $${\text{Overall:}}\;{\text{H}}_{2} {\text{O}} + {\text{CO}}_{2} \to {\text{HCOOH}} + 0.5{\text{O}}_{2} \quad \Delta G^{0} = 270.139\;{\text{kJ}}\;{\text{mol}}^{{ - 1}}$$

18 $$E^{0} = 1.40 \;V$$

### Calculation of Energy Efficiency (EE)

EE of CO_2_RR//FOR electrolyzer is calculated as follows:

19 $${\text{EE}} = \frac{{{\text{Energy }}\;{\text{required}}}}{{{\text{Energy}}\;{\text{ input}}}} = \frac{{\left| {\Delta G^{\theta } } \right|n}}{{E_{{{\text{cell}}}} It}} = \frac{{z|E^{\theta } |Fn}}{{E_{{{\text{cell}}}} It}} = \frac{{\left| {E^{\theta } } \right|Q}}{{E_{{{\text{cell}}}} It}} = \frac{{|E^{\theta } |it}}{{E_{{\text{ cell}}} it}} \times {\text{FE}} = \frac{{|E^{\theta } |}}{{E_{{{\text{cell}}}} }} \times {\text{FE}}$$

where Δ*G*^0^ (kJ mol^−1^) is the standard Gibbs free energy, *n* (mol) is the moles of the desired product, Δ*E*^0^ (*V*) is the thermodynamic equilibrium potential for a specific product, *F* is Faraday constant (96,485 C mol^−1^), *z* is the number of transfer electron, *Q* is the total amount of charge transferred into the desired product, *i* (A) is the total current density, *t* (*s*) is the electrolysis time, and *E*_cell_ (*V*) is the applied cell voltage.

It was calculated for the cathodic CO_2_RR half-cell by assuming the overpotential of the anodic OER which was zero.

20 $${\text{Cathodic}}\; {\text{EE}} = \frac{{E_{{{\text{OER}}}}^{0} - E_{{{\text{CO}}_{2} {\text{RR}}}}^{0} }}{{E_{{{\text{OER}}}}^{0} - E}} \times {\text{FE}}$$

where $E_{{{\text{OER}}}}^{0}$ is the thermodynamic equilibrium potential of OER (1.23 *V*_RHE_), $E_{{{\text{CO}}_{2} {\text{RR}}}}^{0}$ is the thermodynamic equilibrium potential of CO_2_RR to formate (− 0.17 *V*_RHE_), *FE* is the measured formate faradaic efficiency, and *E* is the applied potential (*V*_RHE_).

#### Calculation of Electricity Consumption for Formate Production

Electricity consumption is calculated as follows:

21 $$W = E \times i \times t$$

where *W* is the electricity consumption (Wh), *E* is the cell voltage of CO_2_RR//FOR full cell (*V*), *i* is the current (*A*), and *t* is the electrolysis time (*h*).

For ECR, produced formate is calculated as follows:

22 $$m_{{{\text{formate}}}} = \frac{it}{{2F}} \times {\text{FE}} \times M_{{{\text{formate}}}}$$

For FOR, produced formate is calculated as follows:

23 $$m_{{{\text{formate}}}} = \frac{it}{F} \times {\text{FE}} \times M_{{{\text{formate}}}}$$

where *i* is recorded current (*A*), *t* is reaction time (*s*), and *M*_formate_ is molar mass of formate (45 g mol^−1^).

Electricity consumption normalized by the mass of formate produced in CO_2_RR//FOR full cell:

24 $${\text{Formate}} = \frac{{\text{Electricity consumption}}}{{m_{{{\text{formate}}}} }}$$

## Results and Discussion

### Morphological and Structural Characterization

**Fig. 1:**
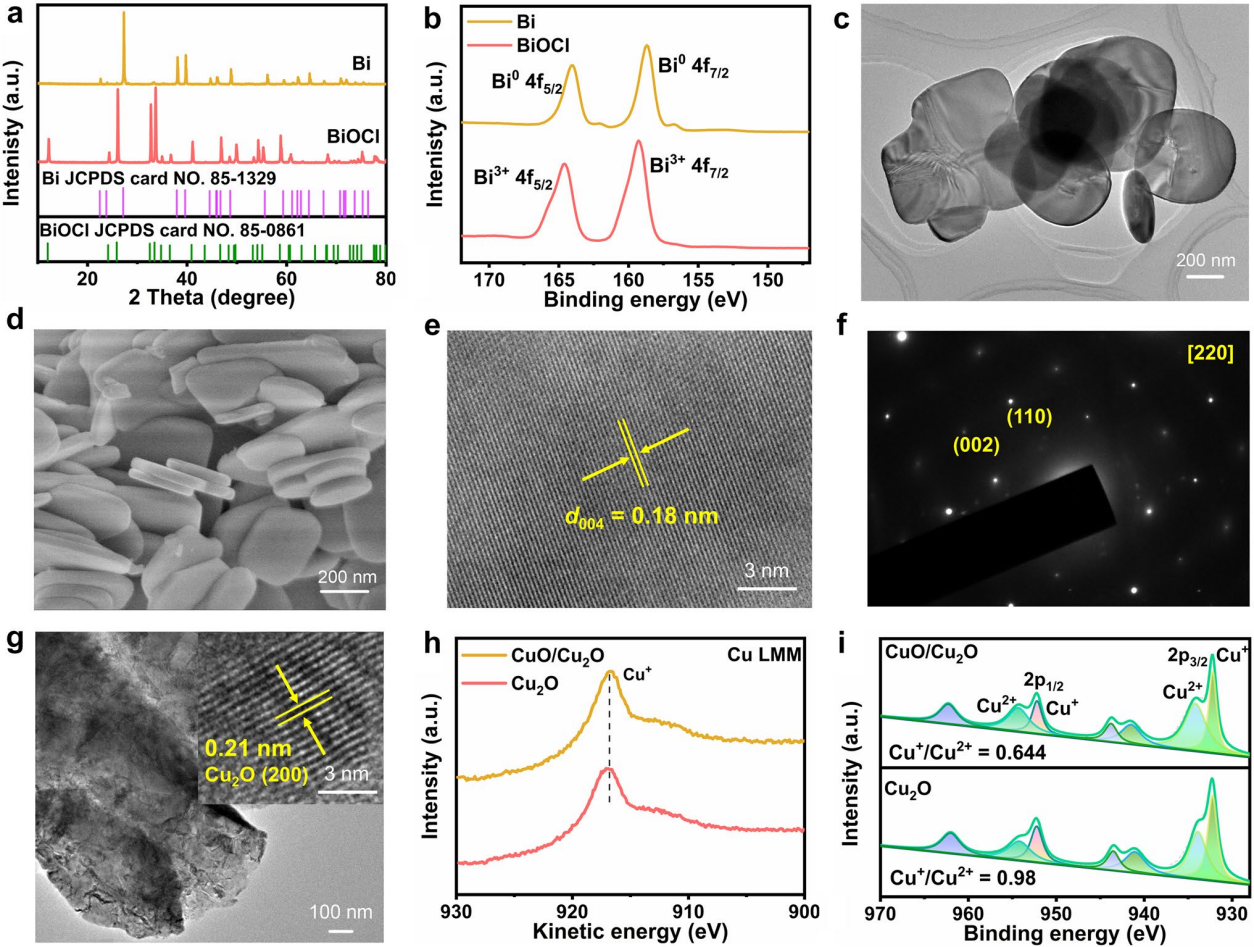
Morphologies and microstructure of CO_2_RR and FOR catalysts. **a** XRD pattern, **b** Bi 4*f* XPS spectra of BiOCl and commercial Bi powder. **c** TEM, **d** SEM, **e** HRTEM images, **f** SAED pattern of BiOCl. **g** TEM and HRTEM images, **h** Auger Cu LMM, **i** Cu 2*p* spectrum of CuO/Cu_2_O and Cu_2_O. (Color figure online)

BiOCl nanosheet was prepared via a simple one-step hydrothermal method. As shown in Fig. 1a, 2*θ* peaks of the commercial Bi powder are in agreement with the rhombohedral Bi (JCPDS No. 85-1329). All diffraction peaks in the XRD pattern of the BiOCl nanosheet are legible and consistent with tetragonal BiOCl (JCPDS No. 85-0861), indicating its high degree of crystallinity. Furthermore, elemental composition and valence state of the commercial Bi powder and the BiOCl nanosheet were confirmed by XPS. Specifically, pair peaks at 159.3 and 164.6 eV verified the existence of Bi^3+^ in the BiOCl nanosheet (Fig. 1b, red curve) [3]. Similar peaks were found in the XPS spectrum of the commercial Bi powder, as indicated by peaks at 158.7 and 164.0 eV, which are slightly deviated from those in BiOCl nanosheet due to inevitable oxidation to air exposure. The small bumps adjacent to those of Bi^3+^ are assigned to Bi^0^ (156.7 and 162.1 eV); this is in line with what has been found in the previous work [28]. The morphology of the obtained BiOCl was characterized by SEM and TEM. As shown in Fig. S1, the commercial Bi powder has a particle size of several micrometers, whereas the prepared BiOCl nanosheet has a smooth surface and a thickness of 50 nm (Fig. 1c–d), which will provide enriched active sites for CO_2_RR. The high-resolution TEM (HRTEM) image of the BiOCl nanosheet in Fig. 1e showed clear lattice fringes of 0.18 nm, corresponding to the (004) lattice plane. The selected area electron diffraction (SAED) pattern of the BiOCl nanosheet displayed a set of diffraction spots, indicating that it is close to single crystalline (Fig. 1f) [8]. Its zone axis was indexed along [200] direction.

**Fig. 2:**
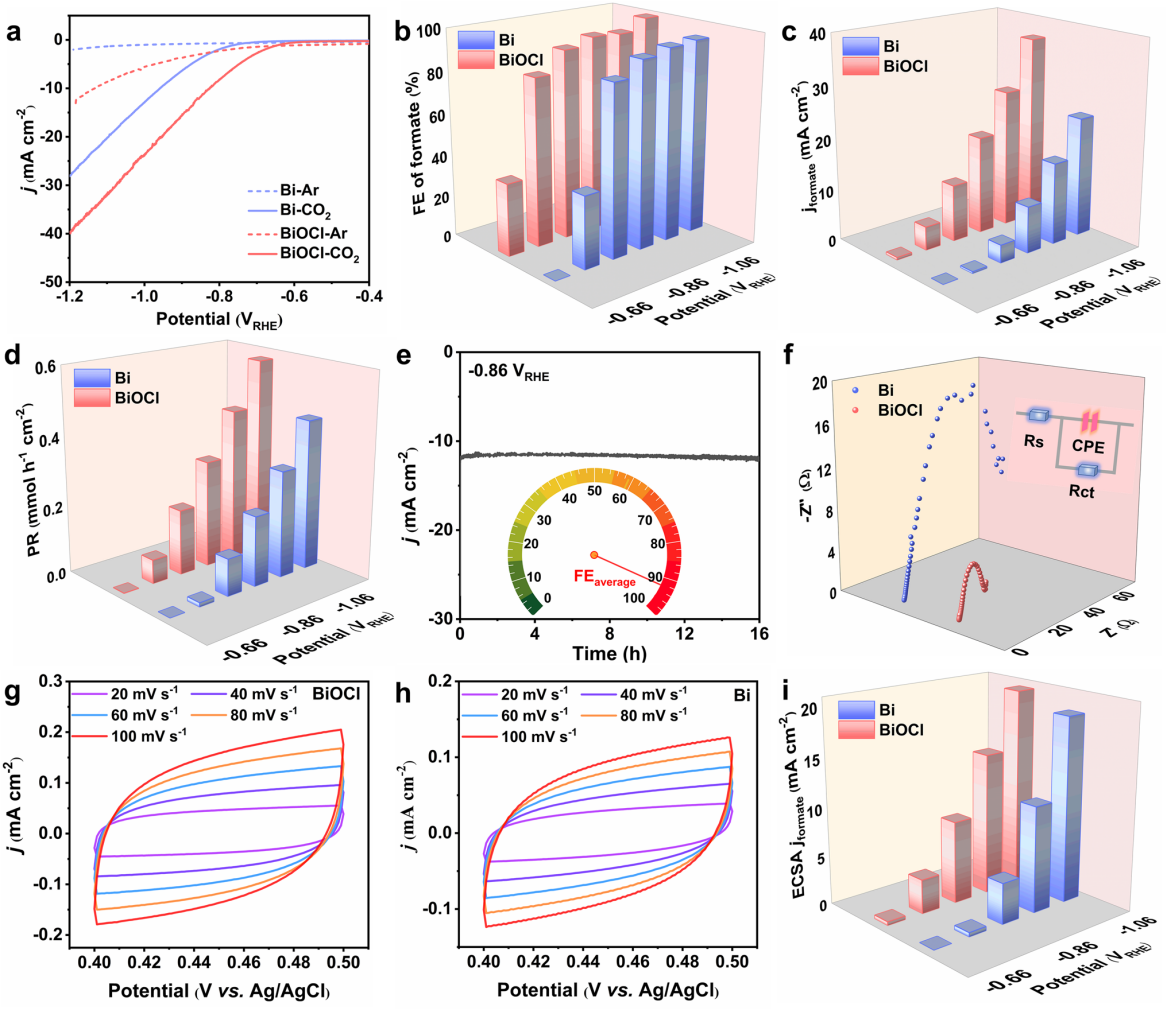
Electrochemical performance of Bi-based catalysts for cathodic CO_2_RR in H-cell. **a** LSV curves of BiOCl and commercial Bi powder in Ar- or CO_2_-saturated 0.5 M KHCO_3_. **b** Formate FE, **c** formate partial current density and **d** formate production rate (PR) of BiOCl and commercial Bi powder at different potentials. **e** Long-term stability test of BiOCl at − 0.86 *V*_RHE_ for 16 h. **f** Nyquist plots of BiOCl and commercial Bi powder at − 0.76 *V*_RHE_ (inset is the corresponding equivalent circuit diagram). CV curve of **g** BiOCl nanosheet and **h** commercial Bi powder at different scan rates. **i** ECSA-corrected formate partial current density

On the other hand, Cu foam was firstly treated with oxygen plasma (denoted as Cu1) and then electrochemically reduced to Cu_2_O (denoted as Cu2). After oxygen plasma treatment, XRD diffraction peaks of Cu1 unfolded the intense signals from Cu foam substrate with those of Cu_2_O and CuO overlaying on the top (Fig. S2). After electrochemical reduction at − 0.4 *V*_RHE_ for 400 s, all diffraction peaks of Cu2 indexed to Cu foam due to the content of Cu_2_O was below the detection threshold of XRD (Fig. S2). HRTEM was performed to further confirm the exact chemical composition of Cu2 (Fig. 1g). HRTEM image revealed that the obtained electrocatalyst Cu2 is Cu_2_O, as indicated by the lattice fringes of 0.21 nm corresponding to the Cu_2_O (200) lattice plane. Furthermore, Auger Cu LMM was performed to distinguish Cu^0^ from Cu^+^ (Fig. 1h), with no evidence of metallic Cu^0^ appearing at the surface of CuO/Cu_2_O and Cu_2_O (916.97 eV related to Cu^+^) [29]. Cu 2*p* XPS spectra of CuO/Cu_2_O and Cu_2_O (Fig. 1i) suggested a mutual existence of Cu^+^ (932.22 and 952.19 eV) and Cu^2+^ (934.18 and 954.31 eV) [30]. After electrochemical reduction at − 0.4 *V*_RHE_ for 400 s, the ratio of Cu^+^–Cu^2+^ increased from 0.644 to 0.980, meaning an increased ratio to Cu^+^. Due to electrochemical reduction of CuO/Cu_2_O performed in 1 M KOH, the electrolyte dissolved oxygen was not removed. Cu is an oxyphilic metal extremely susceptible to oxidation. After electrochemical reduction in 1 M KOH at − 0.4 *V*_RHE_ for 400 s, the current density is about 28 mA cm^−2^ (Fig. S3), indicating that the reduction of CuO/Cu_2_O is not complete.

### Electrocatalytic Performance of Cathodic CO_2_RR

The electrocatalytic performance of BiOCl nanosheet and the commercial Bi powder was measured in an H-cell. The observed current density in CO_2_ was significantly higher than that in Ar-saturated electrolyte, indicating BiOCl and Bi prefer to engender CO_2_RR instead of hydrogen evolution reaction (HER, Fig. 2a). Later, electrolysis was carried out at various potentials between − 0.66 and − 1.16 *V*_RHE_ for 1 h to measure reaction selectivity (Fig. S4). Gas product was analyzed by online GC, and the liquid product was examined by HPLC. Standard curve between the known concentration of formate and relative area has an excellent linear relationship (*R*^2^ = 0.99986) as shown in Fig. S5. The only liquid product for CO_2_RR is formate, accompanied by a small portion of H_2_ (Fig. S6). As shown in Fig. 2b, the FE of formate formation on the BiOCl electrode was less than 50% when the potential was lower than − 0.66 *V*_RHE_. It quickly rose to 81% at − 0.76 *V*_RHE_ and remained above 90% between − 0.86 and − 1.16 *V*_RHE_. It is obvious that formate FE on BiOCl is higher than that on commercial Bi powder within the measured potential range. In addition, formate partial current density and PR on the BiOCl nanosheet and the commercial Bi powder were calculated and plotted against the applied potentials, as shown in Fig. 2c–d. It is self-evident that the CO_2_RR performance on the BiOCl nanosheet is superior to that on the commercial Bi powder. The formate partial current density and PR of BiOCl nanosheet were found to be 36.24 mA cm^−2^ and 0.577 mmol h^−1^ cm^−2^ at − 1.16 *V*_RHE_, much higher than those of the commercial Bi powder (24.22 mA cm^−2^, 0.439 mmol h^−1^ cm^−2^) as well as most of the previously reported catalysts (Table S2). EE is the percentage of energy reserved as chemical energy in a targeted product in the applied electrical energy. The high formate FE and the low overpotential of BiOCl nanosheet contribute to a high EE of over 55% in a wide potential window ranging from − 0.76 to − 1.16 *V*_RHE_; a maximum value of 60.7% is achieved at − 0.86 *V*_RHE_ (Fig. S7).

As another important factor, the stability of the BiOCl electrocatalyst was measured at − 0.86 *V*_RHE_ for 16 h. A stable cathodic current density of 12 mA cm^−2^ and an average formate FE of 92% were observed without decay, suggesting its excellent stability (Fig. 2e). Simultaneously, the BiOCl nanosheet exhibits excellent stability at a higher potential of − 1.16 *V*_RHE_ (Fig. S8). Afterward, the morphology and chemical composition of the BiOCl nanosheet after stability measurement were investigated. The SEM and TEM images in Fig. S9 and S10a–b revealed that BiOCl nanosheet was transformed into nanoparticles with a size between 20 and 100 nm. Discontinuous diffraction rings in the SAED pattern of BiOCl after the stability measurement correspond to rhombohedral metallic Bi (Fig. S10d). Furthermore, diffraction peaks ascribed to metallic Bi were observed in XRD pattern of the tested BiOCl (Fig. S11a). Also, XPS spectrum of Bi 4f for BiOCl after CO_2_RR showed peak pairs at 156.67 and 161.94 eV, which is attributed to Bi^0^ (Fig. S11b). As shown in Fig. S10c, the BiOCl electrocatalyst after CO_2_RR has a blurry lattice fringe and displays a localized disordered structure due to lattice oxygen and chlorine release during the process of CO_2_RR [16]. All those above results demonstrated that BiOCl nanosheet was transferred to metallic Bi under the reduction condition, where Bi is the real active site for CO_2_RR [8, 13, 14, 16].

To investigate the reason for the superior CO_2_RR performance on the BiOCl nanosheet, a set of electrochemical characterizations was performed. To start with, EIS was recorded at − 0.76 *V*_RHE_ from 0.01 Hz to 100 kHz to get a deep understanding of reaction kinetics. Figure 2f shows that BiOCl has a smaller charge-transfer resistance (*R*_ct_) of 15.30 Ω thereby a higher conductivity than that of commercial Bi powder (67.73 Ω), therefore enabling faster charge transfer. The smaller *R*_ct_ lowers the barrier of electron transfer and facilitates the formation of reaction intermediates in CO_2_RR. Furthermore, Fig. 2g–h shows the ECSA of the catalysts determined by CV at different scan rates. ECSA of the BiOCl nanosheet (1.30 mF cm^−2^) is higher than that of the commercial Bi powder (0.834 mF cm^−2^) in Fig. S12. Formate partial current densities of the BiOCl nanosheet normalized by ECSA were also higher than those of the commercial Bi powder, suggesting that BiOCl possesses higher intrinsic activity (Fig. 2i). The superior CO_2_RR performance on the BiOCl nanosheet compared to commercial Bi powder can be attributed to its higher conductivity, more abundant active sites, high intrinsic activity, and the formation of atomic-scale disordering.

**Fig. 3:**
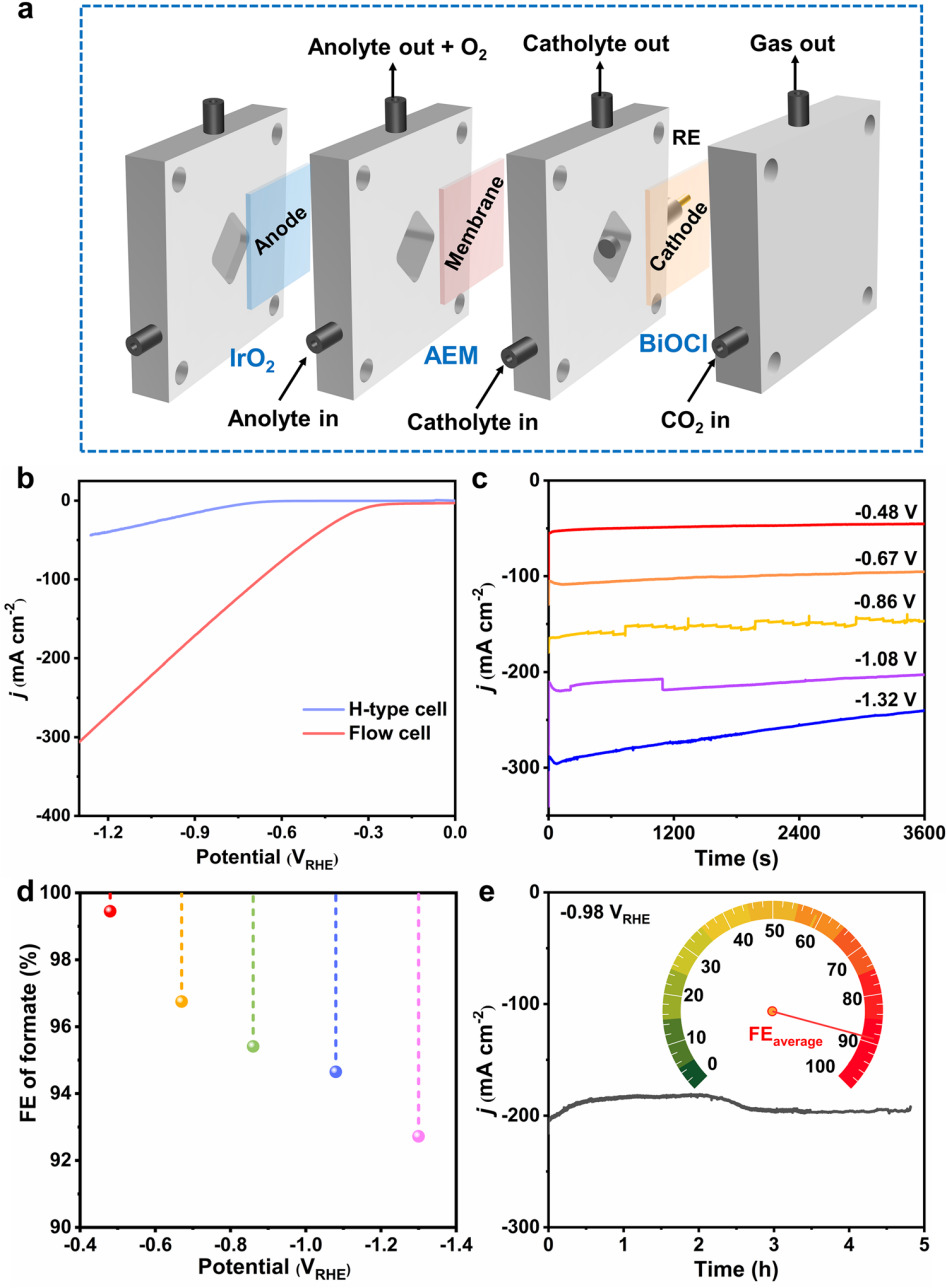
Electrochemical performance of BiOCl for cathodic CO_2_RR in a flow cell. **a** Schematic diagram of flow cell. **b** LSV curves of BiOCl in H-cell and flow cell. **c** Constant potential electrolysis. **d** FE of formate in a wide potential window ranging from − 0.48 to − 1.32 *V*_RHE_. **e** Stability test of BiOCl in flow cell at − 0.98 *V*_RHE_

It has been reported that CO_2_RR ought to reach a FE higher than 95% and a minimum current density of 200 mA cm^−2^ for practical application [13, 31, 32]. In fact, due to the limited solubility of CO_2_ in aqueous electrolyte (34 mmol L^−1^ at 25 °C and 101.325 kPa) [33], the current density of the cathodic CO_2_RR in H-cell is restrained. Therefore, flow cell combined with GDE was used in CO_2_RR to break mass transfer limitation of CO_2_ (Fig. 3a), which allows CO_2_ to react in the form of gas instead of being dissolved in the electrolyte, thus improving the reactivity of the CO_2_RR. On the other hand, using KOH as the supporting electrolyte can suppress the competing HER and lower the CO_2_RR activation energy barrier [13, 34]. As shown in Fig. 3b, the onset potential of BiOCl (− 0.25 *V*_RHE_) in flow cell was less negative than that in H-cell (− 0.65 *V*_RHE_), and the current density at − 1.0 *V*_RHE_ in flow cell (204.75 mA cm^−2^) was 8 times higher than that in H-cell (23.85 mA cm^−2^). The possible reason could be that GDE shortens the diffusion distance of CO_2_ molecules and loads itself with sufficient gaseous reactant [35], and the presence of KOH aqueous solution as electrolyte leads to standard redox potential of CO_2_RR to formate is more positive than that of HER [7]. The product analysis result shows that formate FE on the BiOCl nanosheet in flow cell is higher than 90% between − 0.48 and − 1.32 *V*_RHE_ (Fig. 3c–d). BiOCl nanosheet EE decreases with the applied potentials increment from − 0.48 to − 1.32 *V*_RHE_ in the flow cell (Fig. S13); a maximum value of 81.4% is achieved at − 0.48 *V*_RHE_. To verify the carbon source of formate in the flow cell, Ar-saturated 1 M KOH is used as catholyte. Formate was not detected after electrolysis in Ar-saturated 1 M KOH (Fig. S14), indicating that the carbon source of the formate indeed derives from the CO_2_ used. The SPCE of CO_2_ toward formate using BiOCl nanosheet increases with the increment in the applied potentials from − 0.48 to − 1.32 *V*_RHE_; the highest SPCE is 8.57% at − 1.32 *V*_RHE_ (Fig. S15). In addition, no apparent decline in the current density was observed during the process, demonstrating excellent electrochemical stability (Figs. 3e and S16–S17). Besides, the partial current density of formate on the BiOCl electrode was as high as 219.3 mA cm^−2^ with a FE of 95% at − 1.08 *V*_RHE_ in flow cell, which is the highest among previously reported electrocatalysts (Table S3).

### Electrocatalytic Performance of Anodic FOR

**Fig. 4:**
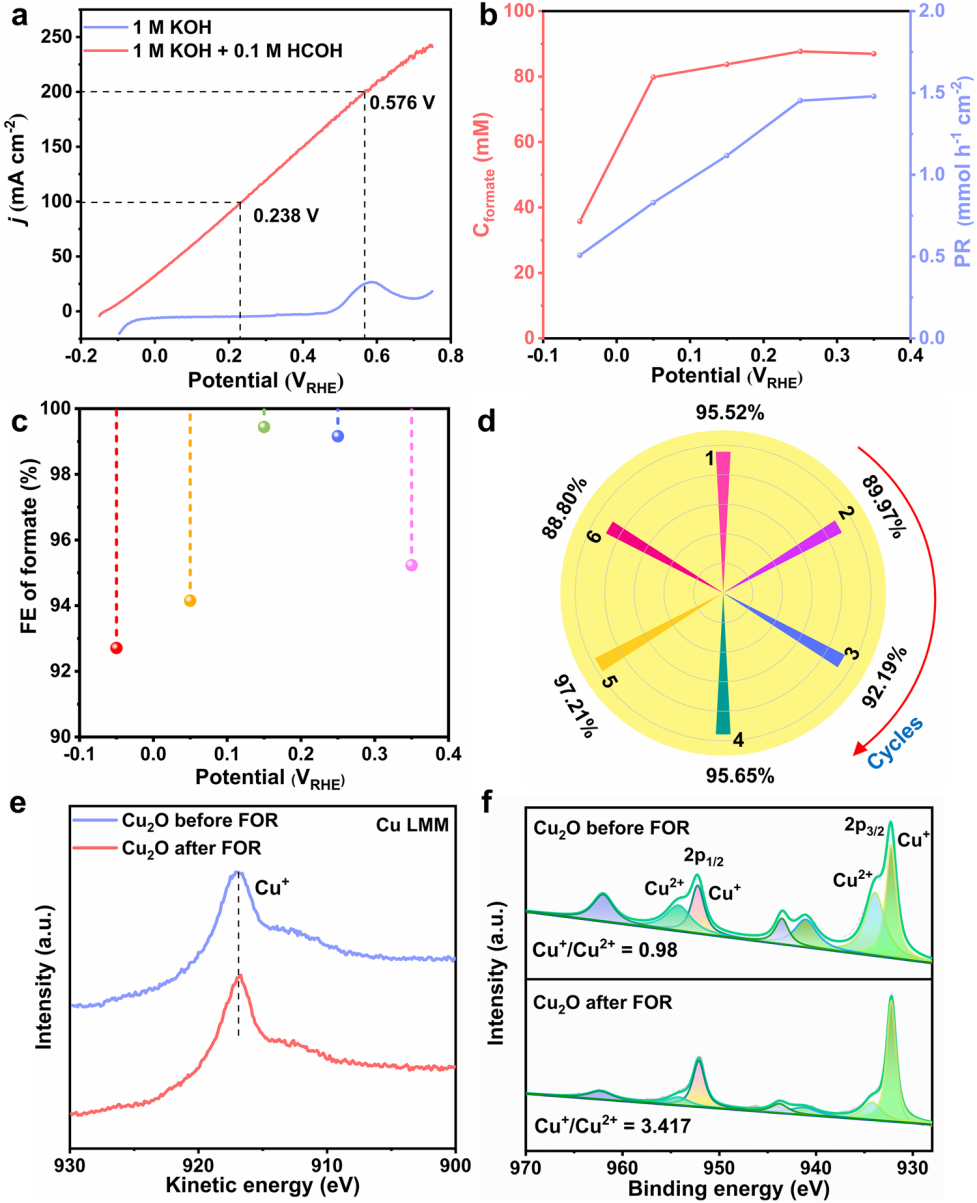
Electrochemical performance and structural characterization of Cu_2_O for anodic FOR. **a** LSV curves of Cu_2_O in 1 M KOH electrolyte with or without formaldehyde. **b F**ormate concentration formed by formaldehyde electrooxidation and total formate production rate (PR), **c** FE of formate for complete conversion of formaldehyde at different potentials. **d** FE of formate using Cu_2_O for six successive electrolysis cycles. **e** Auger Cu LMM, **f** Cu 2*p* spectrum of Cu_2_O before and after FOR

Compared with OER, FOR on Cu_2_O electrode is thermodynamically favored, demonstrated by the onset potential of − 0.13 *V*_RHE_ in the LSV curves (Fig. 4a). It requires as low as 0.238 *V*_RHE_ to reach the current density of 100 mA cm^−2^, indicating its excellent electrocatalytic activity. Electrooxidation of formaldehyde is accompanied by the evolution of hydrogen and formate formation (HCOH + OH^−^ − e^−^ → HCOOH + 1/2 H_2_). This single electron transfer process is verified by the linear relationship between the formate concentration and consumed charge (Fig. S18). EIS was carried out from − 0.25 to − 0.05 *V*_RHE_ to understand reaction kinetics (Fig. S19). It turned out that *R*_ct_ experienced a gradual increase from − 0.25 to − 0.15 *V*_RHE_, indicating the reduction of Cu_2_O is increasingly difficult to occur. In contrast, *R*_ct_ gradually decreased from − 0.15 to − 0.05 *V*_RHE_ due to the occurrence of the FOR. The summary of the corresponding fitting data of Cu_2_O at different potentials is presented in Table S4.

MOR and OER performance of Cu_2_O were tested in 1 M KOH with or without the addition of 0.1 M methanol to verify the effect of methanol on the performance of formaldehyde oxidation. It can be observed that the current density is almost unchanged after the addition of 0.1 M methanol to 1 M KOH electrolyte in Fig. S20, indicating that the MOR performance of Cu_2_O is poor. Thus, the current density of Cu_2_O in 1 M KOH electrolyte containing 0.1 M formaldehyde is derived from FOR rather than MOR or OER. The presence of methanol does not affect on the performance of formaldehyde oxidation.

Electrolysis was performed at different potentials from − 0.05 to 0.35 *V*_RHE_. Liquid product was analyzed by NMR at the end of electrolysis (Fig. S21), while the gas product was analyzed by online GC. Figure 4b presents the concentration and production rate of formate as a function of the applied potentials. The highest formate concentration formed by formaldehyde electrooxidation was 87.7 mM at 0.25 *V*_RHE_. In addition, there is 10.1 mM formate (Fig. S22) derived from the Cannizzaro reaction. (Aldehyde without α-hydrogen atom undergoes an intermolecular redox reaction under strong base electrolyte to form a carboxylic acid and an alcohol.) [36] The production rate of formate increased with the increment of the applied potentials. As the potential increases, the time required for the complete conversion of formaldehyde is shortened. When electrolysis was performed at 0.35 *V*_RHE_, formate production rate was 1.48 mmol h^−1^ cm^−2^. Furthermore, FE of formaldehyde electrochemical oxidation to formate and H_2_ was higher than 90% within the potential ranging from − 0.05 to 0.35 *V*_RHE_ (Figs. 4c and S23). Formaldehyde electrooxidation performance catalyzed by Cu_2_O at different potentials is listed in Table S5. A stability test was carried out at 0.35 *V*_RHE_ for the complete conversion of formaldehyde to evaluate the durability of Cu_2_O (Fig. S24). The concentration of formaldehyde and the current density gradually decreased with the prolongation of electrolysis time. When formaldehyde is completely converted, the current density closes to zero. The electrolyte was replaced with a freshly prepared one with formaldehyde to test the next cycle. The experiments were carried out 6 times. As shown in Fig. 4d, the formate FE was almost 90% for six successive electrolysis cycles, formate moles, and production rate without a noticeable decrease (Fig. S25), indicating outstanding stability.

To investigate the superior catalytic performance of the Cu_2_O catalyst, Auger Cu LMM and Cu 2*p* spectra were performed after FOR at 0.35 *V*_RHE_ (Fig. 4e–f). Auger Cu LMM spectrum revealed the existence of Cu^+^ rather than Cu^0^ (916.73 eV related to Cu^+^). Moreover, the ratio of Cu^+^ to Cu^2+^ increased from 0.980 to 3.417. On the one hand, reduction of Cu^2+^ by H_2_ generated from the electrooxidation of formaldehyde. On the other hand, spontaneous reaction between aldehyde groups and Cu^2+^ in strong alkaline electrolyte forms Cu_2_O and carboxylic acid [37]. CV curve of Cu_2_O is shown in Fig. S26, and oxidation peak from 0.5 to 0.7 *V*_RHE_ in 1 M KOH electrolyte is assigned to Cu/Cu^+^. As a result, FOR was performed at potentials from − 0.05 to 0.35 *V*_RHE_, prior to the oxidation of Cu^+^/Cu^2+^, and Cu_2_O is stable in a wide potential window. To verify the role of Cu^0^, a piece of 1 cm^2^ Cu foam acts as catalyst for FOR. As expected, Cu foam has almost no FOR performance (Fig. S27). Surprisingly, after the first and the second LSV from − 0.35 to 1.05 *V*_RHE_, the copper foam is first oxidized and then electrochemically reduced, exhibiting excellent FOR performance different from the initial Cu foam. However, all the above results show that the tremendous catalytic performance of Cu_2_O probably originates from Cu^+^. In short, Cu_2_O is an efficient and highly selective catalyst for FOR.

### Electrochemical Performance of CO_2_RR Coupled with FOR

Owing to the remarkable CO_2_RR performance on the BiOCl nanosheet and excellent FOR performance on Cu_2_O, a two-electrode electrolyzer was constructed. In H-cell, CO_2_-saturated 0.5 M KHCO_3_ and 1 M KOH with 0.1 M HCOH were used as catholyte and anolyte, respectively. The catholyte is neutral, and the anolyte is alkaline; formic acid is the primary product of cathodic CO_2_RR; PEM acts as a separator to separate the cathode and the anode because formic acid can balance the transport of metal cations from anode to cathode and form corresponding metal salts [35]. Schematic illustration of the two-electrode electrolyzer is presented in Fig. S28a. This electrolyzer requires as low as 1.00 V to reach the current density of 10 mA cm^−2^ (Fig. S28b), which is much lower than that of CO_2_RR//OER (2.60 V). Electrolysis was performed at different voltages between 1.05 and 2.20 V for 2 h (Fig. S28c). The FE of formate and formate production rate for anodic FOR, cathodic CO_2_RR, and CO_2_RR//FOR full cell are presented in Fig. S28d–e. Formate FE of CO_2_RR//FOR full cell maintained higher than 180% from 1.05 to 2.20 V. Formate production rate of CO_2_RR//FOR full cell increased from 1.013 (1.05 V) to 1.679 mmol h^−1^ cm^−2^ (2.20 V). The EE of CO_2_RR//FOR in the H-cell at 1.05 V is 4.82%. All those above measurement results revealed that CO_2_RR coupling with FOR is an efficient strategy for the electrosynthesis of formate.

Although the cell voltage was reduced by coupling CO_2_RR with FOR, the current density of the electrolyzer is limited by the mass transport of CO_2_, large ohmic resistance, long CO_2_ diffusion distance, and the poor CO_2_RR catalytic activity in the near-neutral electrolyte (CO_2_-saturated 0.5 M KHCO_3_). Thus, CO_2_RR//FOR was constructed in a liquid-phase flow cell combined with GDE in strong alkaline electrolyte, providing a triple-phase interface for CO_2_ to contact the catalyst–electrolyte interface to break the mass transfer limits of CO_2_ (Fig. S29). In the flow cell, KOH and KOH containing 0.1 M HCOH were used as catholyte and anolyte, respectively. Since catholyte and anolyte are alkaline, AEM acts as a separator to separate the cathode and the anode, and the distance between the cathode and anode is about 2 cm. Enhanced current density (100 mA cm^−2^) was observed for the CO_2_RR//FOR full cell with the aforementioned design, which is nine times higher than that in H-cell at the same potential of 1.21 V (Fig. S30). Electrolysis was performed at different voltages between 0.6 and 1.5 V (Figs. S31a and S32a). Besides, the total formate FE consisting of the anodic FOR and cathodic CO_2_RR remained above 160% in a wide voltage ranging from 0.6 to 1.5 V (Figs. S31b and S32b). The EE of CO_2_RR//FOR in the flow cell at 0.6 V is 7.92%.

**Fig. 5:**
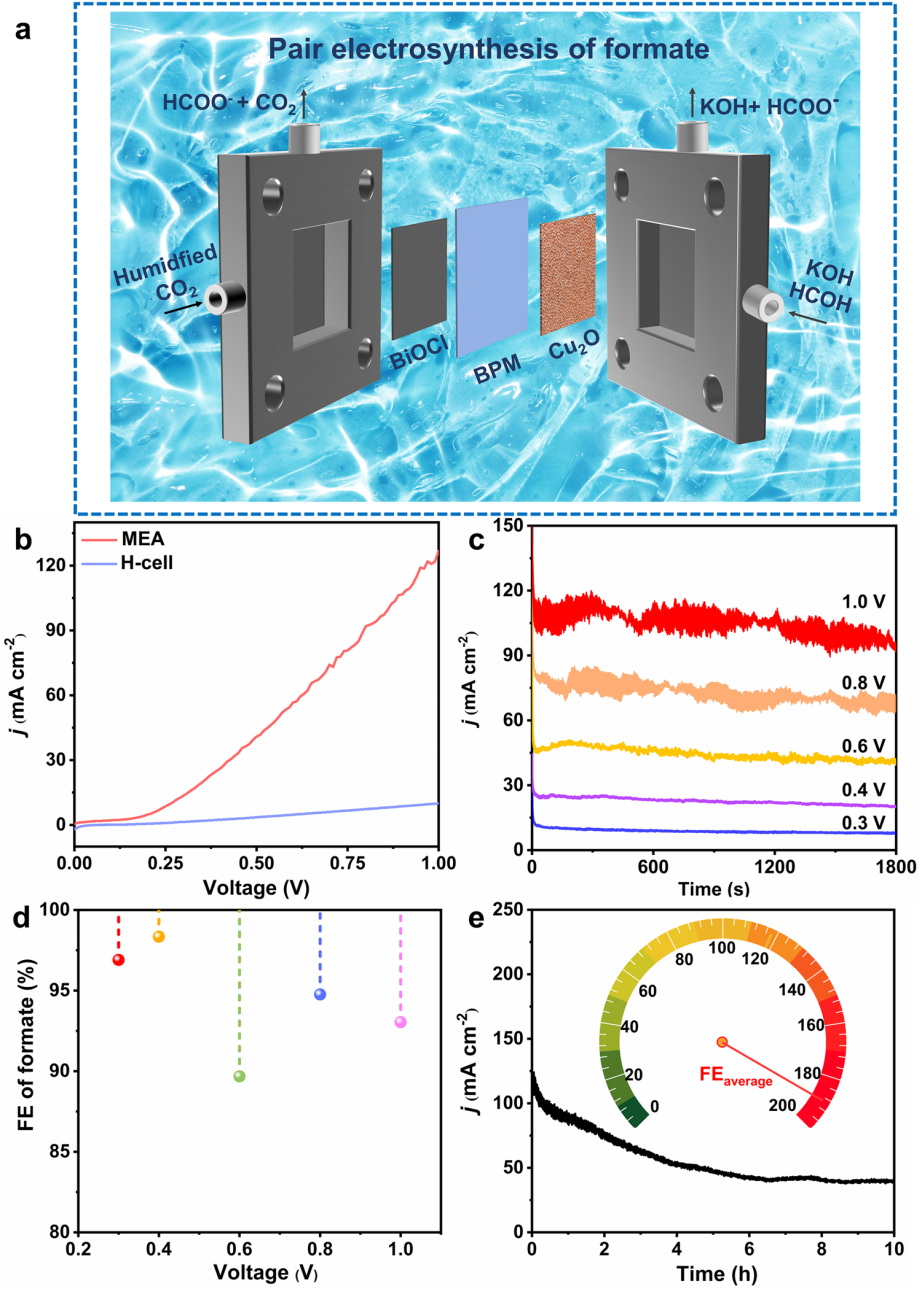
Electrochemical performance of CO_2_RR coupled with FOR in MEA. **a** Schematic diagram for CO_2_RR//FOR in a gas-phase flow cell with MEA. **b** LSV curves of CO_2_RR//FOR in H-cell and MEA. **c** Constant potential electrolysis, **d** voltage-dependent formate FE range from 0.3 to 1.0 V. **e** Long-term stability test at 1.0 V for 10 h

Up to date, MEA has not yet been applied to formate pair-electrosynthesis. The MEA electrolyzer is the state-of-the-art reaction platform for CO_2_RR at industrially relevant scales [38]. MEA with a unique zero-gap configuration could shorten the distance between the anode and the cathode from several centimeter to a few tens of micrometers (Fig. 5a), distinctly decreasing the internal resistance and ohmic loss as well as avoiding GDE flooding, resulting in enhanced system stability [35]. In MEA, pre-humidified CO_2_ flows into the cathode plate to keep the ion exchange membrane moist, and 1 M KOH containing 0.1 M HCOH was used as anolyte. Since AEM is only 25 μm, the uneven surface of the three-dimensional copper foam substrate will puncture the membrane causing short circuits and electrolyte leakage. On the other hand, the flow rate of the anolyte is 133.9 sccm; such a fast flow rate can lead to damage to AEM. BPM acts as a solid electrolyte in MEA, and the distance between the cathode and the anode is about 130 μm. The low onset potential of CO_2_RR//FOR cell is that kinetic-sluggish OER at the anode is replaced by thermodynamically more favorable FOR, drastically reducing energy consumption. FOR equilibrium potential is − 0.224 *V*_RHE_ (2HCOH + 2OH^− ^− 2e^−^ → 2HCOOH + H_2_), significantly lower than OER (1.23 *V*_RHE_, 2H_2_O → 4H^+^ + 4e^−^ + O_2_. Cathodic CO_2_RR to formate migrates to the anolyte under the action of the electric field, providing an opportunity to collect concentrated liquid product formate at the anode. We directly detected formate in the anolyte and calculated the average formate FE of the cathodic CO_2_RR and anodic FOR based on the amounts of total formate moles. It turned out that CO_2_RR//FOR in MEA requires cell voltage as low as 0.86 V to reach the current density of 100 mA cm^−2^ (Fig. 5b), much lower than most of the reported CO_2_RR conversion systems [19, 39]. Figure 5c indicates that the CO_2_RR//FOR full cell in MEA possesses stable chronoamperometry responses at various constant voltages, and the formate FE was found to be as high as 93% at 1.0 V (Fig. 5d). The EE of CO_2_RR//FOR in MEA at 0.3 V is 17.4%. The durability test of the CO_2_RR//FOR electrolyzer was performed at 1.0 V for 10 h, and the result is shown in Fig. 5e. During the long-term electrolysis, the current density of CO_2_RR//FOR cell gradually decreases due to the decrease in the formaldehyde concentration in the anolyte. Also, carbonate deposits on the flow field and the backside of the GDE suppress CO_2_ mass transport and block the active site of the catalyst. The CO_2_RR//FOR electrolyzer exhibited excellent formate electrosynthesis performance with an average formate FE of 192% in MEA. During the 10-h electrolysis, electricity consumption normalized by the mass of formate produced in CO_2_RR//FOR at 1.0 V was 0.413 Wh g^−1^, much higher than that of CO_2_RR//MOR (2.61 Wh g^−1^) [40]. It is obvious that CO_2_RR//FOR combination demonstrates in MEA itself as a promising alternative for the pair-electrosynthesis of formate (Table S6).

## Conclusion

In this work, an efficient strategy was developed to realize pair-electrosynthesis of formate at ultra-low voltage. The CO_2_ reduction reaction (CO_2_RR) was carried out on the BiOCl nanosheet at the cathode, and the formaldehyde oxidation reaction (FOR) occurred on the Cu_2_O electrode at the anode. BiOCl nanosheet displayed a formate FE of 98.98% at − 1.16 *V*_RHE_ with a partial current density of 36 mA cm^−2^. In a flow cell configuration, the current density of over 200 mA cm^−2^ was achieved for CO_2_RR to formate with more than 90% FE within a wide potential range from − 1.08 to − 1.32 *V*_RHE_ as well as excellent stability. On the other side, Cu_2_O enables excellent FOR performance with excellent FE (higher than 90% over a broad potential from − 0.05 to 0.35 *V*_RHE_) and durability (reused for six continuous electrolysis cycles). Finally, CO_2_RR and FOR were constructed in a membrane electrode assembly (MEA) to improve mass transport of CO_2_ and reduce the internal resistance and ohmic loss. Meanwhile, it exhibited excellent performance with a current density of 100 mA cm^−2^ at 0.86 V. The above results provide an efficient way for the preparation of formate at ultra-low voltage, serving as a promising alternative for converting waste resources to valuable chemicals at a low energy cost.

## Supplementary Information

Below is the link to the electronic supplementary material.

Supplementary file1 (PDF 2778 KB)
