# Supplementary material for: A Pair-Electrosynthesis for Formate at Ultra-Low Voltage Via Coupling of CO2 Reduction and Formaldehyde Oxidation
Source: Nanomicro Lett. 2022 Nov 1;14:211. doi: 10.1007/s40820-022-00953-y (PMC9626726; doi:10.1007/s40820-022-00953-y)
Supplement: Supplementary file 1 — Supplementary file1 (PDF 2778 KB) [file 40820_2022_953_MOESM1_ESM.pdf]

Supporting Information for

A Pair-Electrosynthesis for Formate at Ultra-Low Voltage via Coupling of CO_2 Reduction and Formaldehyde Oxidation

Mengyu Li[1], Tehua Wang[1], Weixing Zhao[1], Shuangyin Wang[1], Yuqin Zou[1, 2, *]

[1]State Key Laboratory of Chemo/Bio-Sensing and Chemometrics, College of Chemistry and Chemical Engineering, Advanced Catalytic Engineering Research Center of the Ministry of Education, Hunan University, Changsha 410082, P. R. China

[2]School of Chemistry and Chemical Engineering, Jishou University, Jishou Human 416000

*Corresponding author. E-mail: yuqin_zou@hnu.edu.cn (Yuqin Zou)

Supplementary Figures and Tables

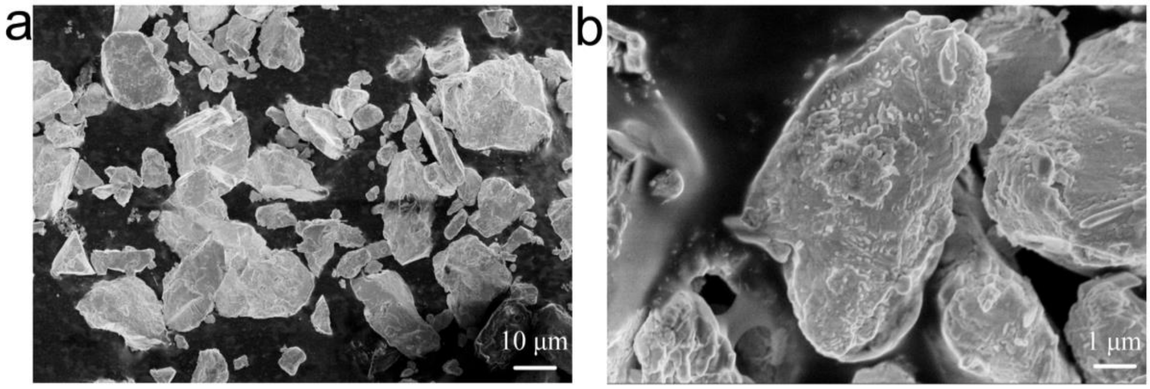

Fig. S1 SEM images of commercial Bi powder at different magnifications

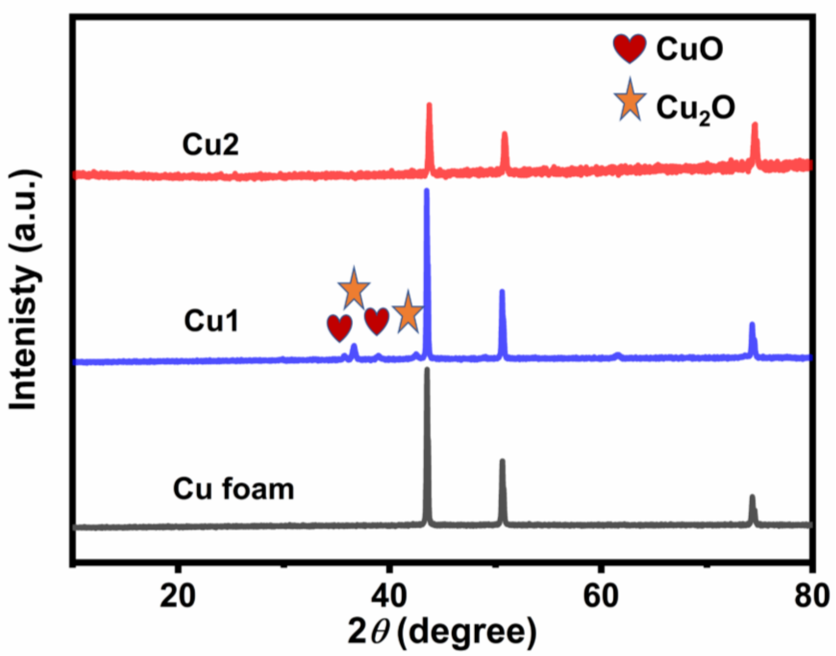

Fig. S2 XRD patterns of Cu foam, Cu1 and Cu2

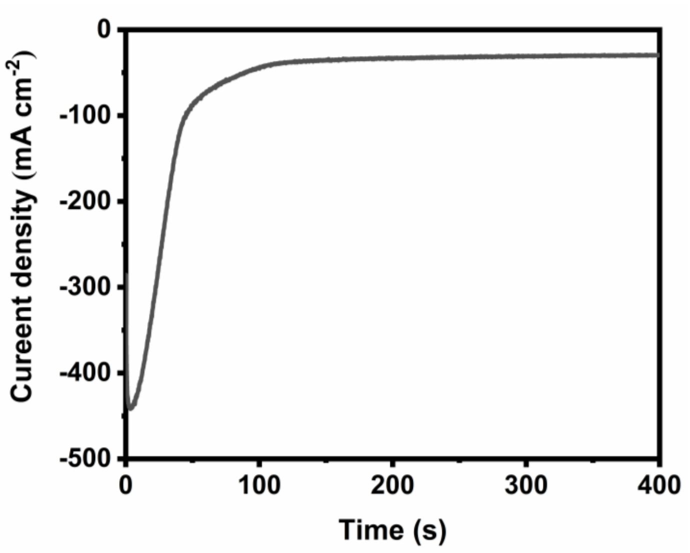

Fig. S3 Electrochemical reduction curve of CuO/Cu_2O at -0.4 V_{RHE} for 400 s in 1 M KOH electrolyte

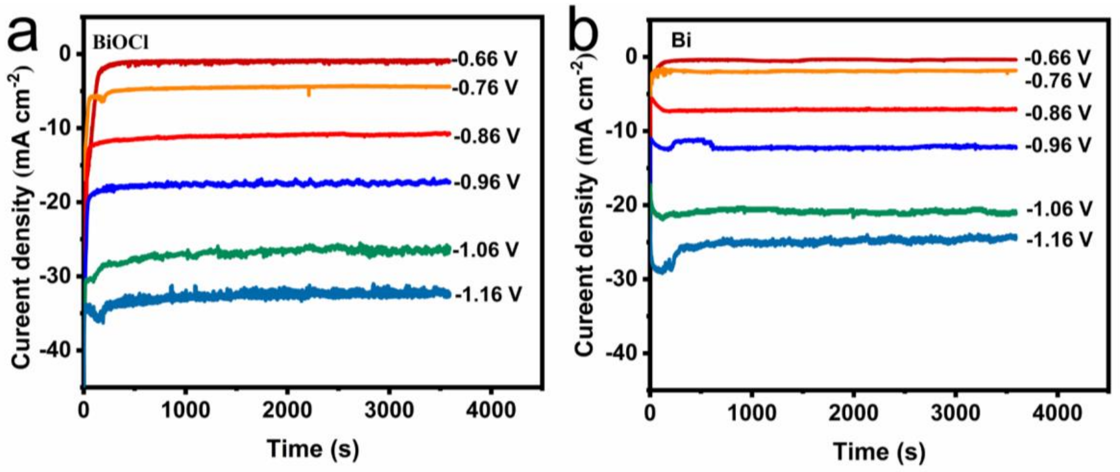

Fig. S4 Constant potential electrolysis of (**a**) BiOCl and (**b**) commercial Bi powder at the different potentials in H-cell

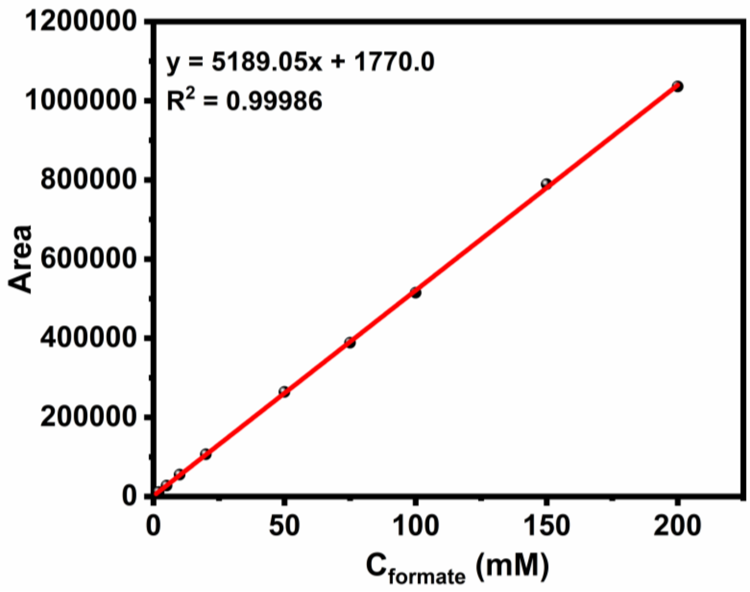

Fig. S5 Standard curve between the known concentration of formate and relative area measured by HPLC

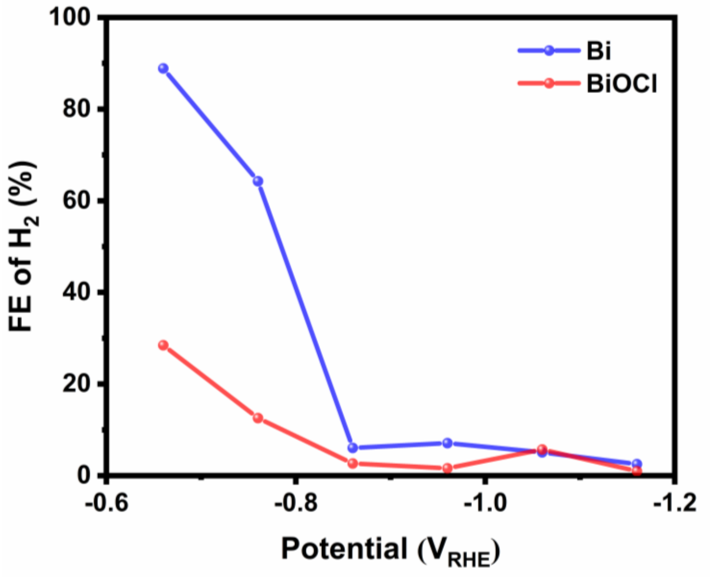

Fig. S6 Potential-dependent FE of CO_2 reduction gas product for BiOCl and commercial Bi powder

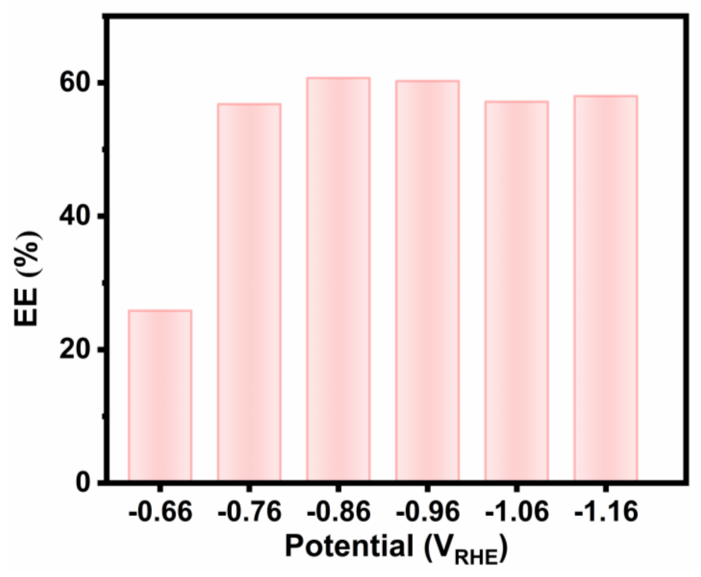

Fig. S7 EE of BiOCl nanosheet for cathodic CO_2RR in H cell

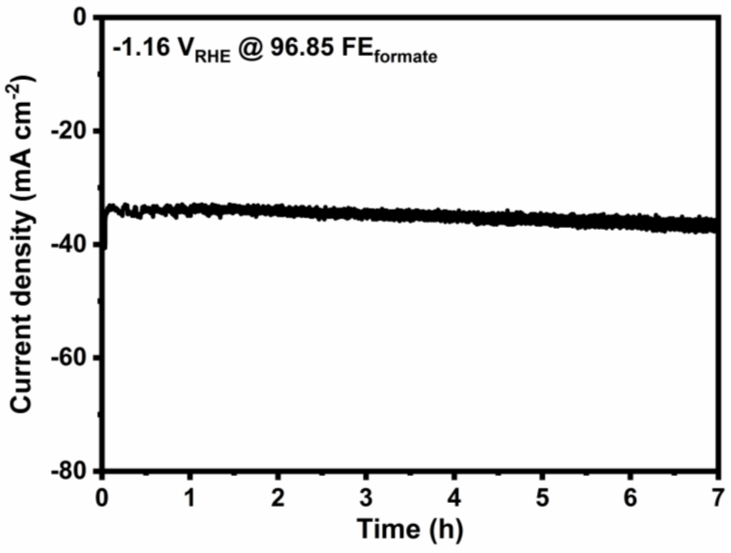

Fig. S8 Long-term stability test of BiOCl nanosheet at -1.16 V_{RHE} in H-cell

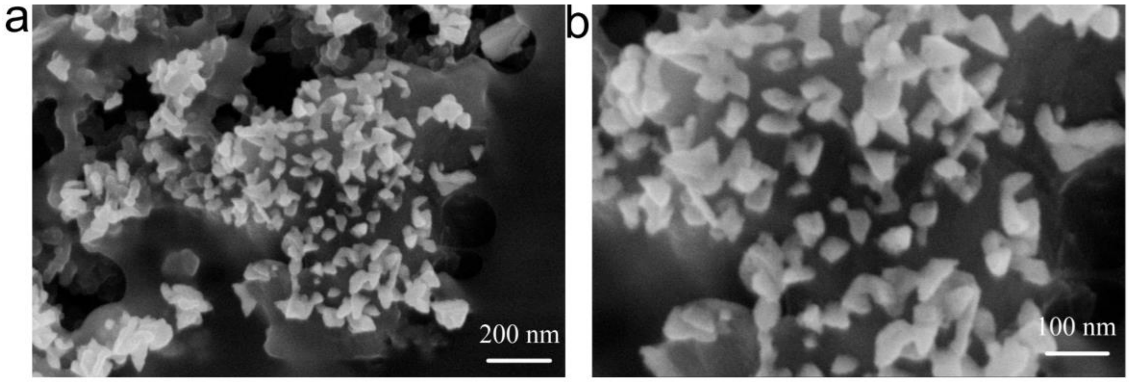

Fig. S9 SEM images of BiOCl nanosheet after CO_2RR

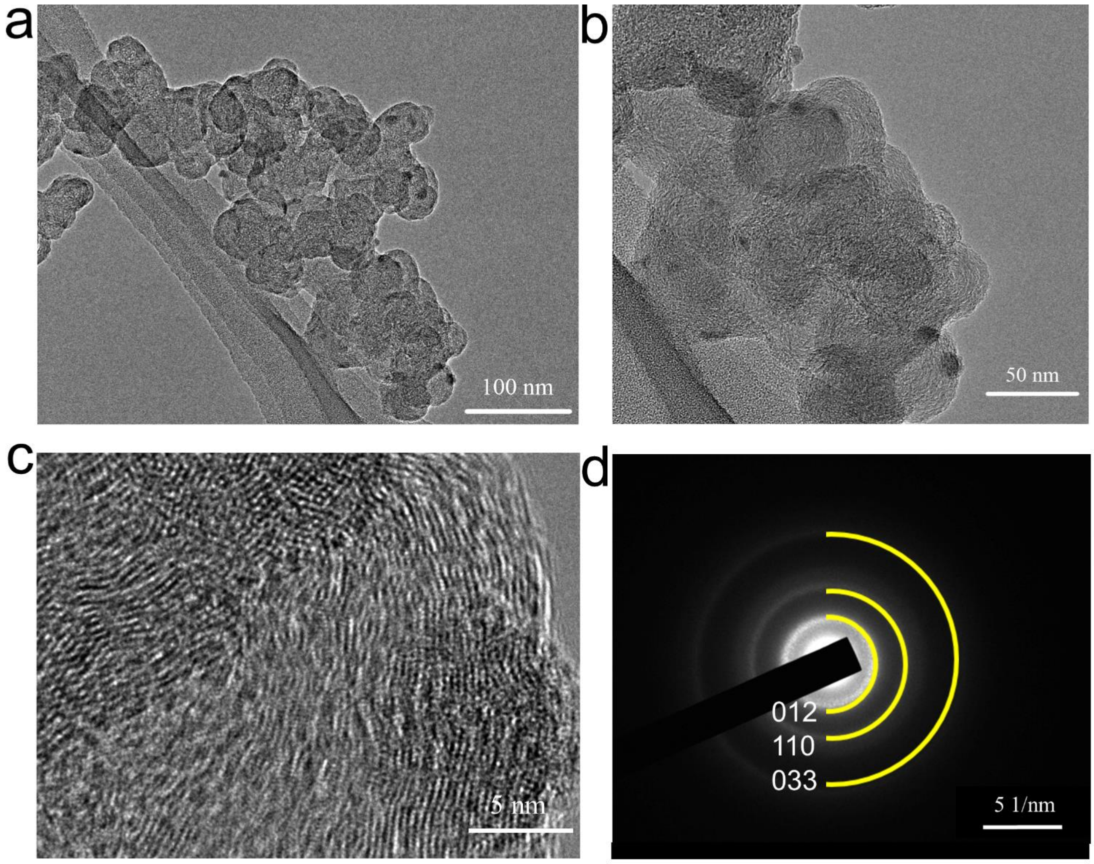

Fig. S10 (**a, b**) TEM images, (**c**) HRTEM image, (**d**) SAED pattern of BiOCl nanosheet after CO_2RR

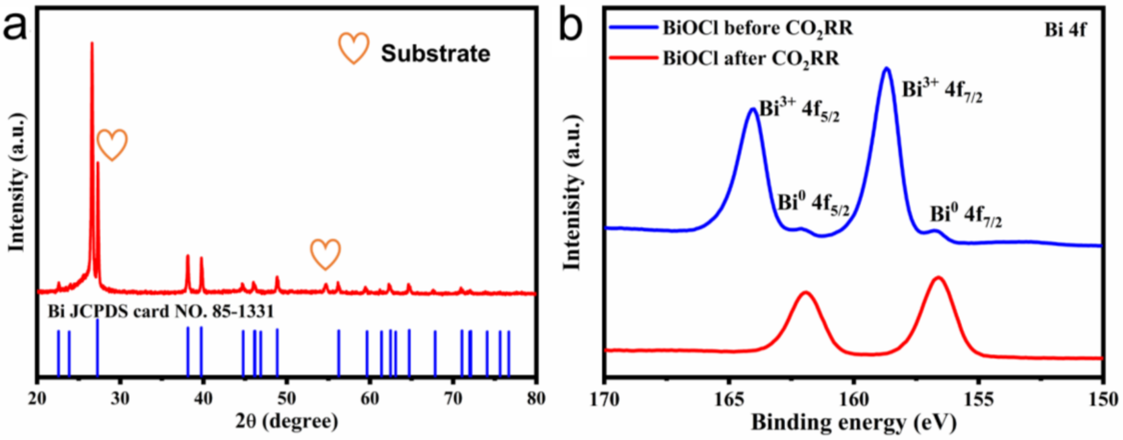

Fig. S11 (**a**) XRD pattern and (**b**) Bi 4f XPS spectrum of BiOCl nanosheet after CO_2RR

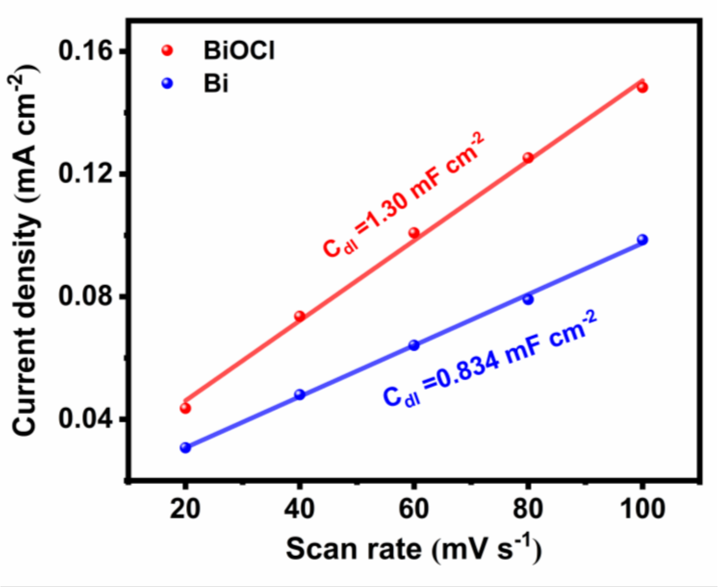

Fig. S12 The capacitive current density differences plotted against scan rates

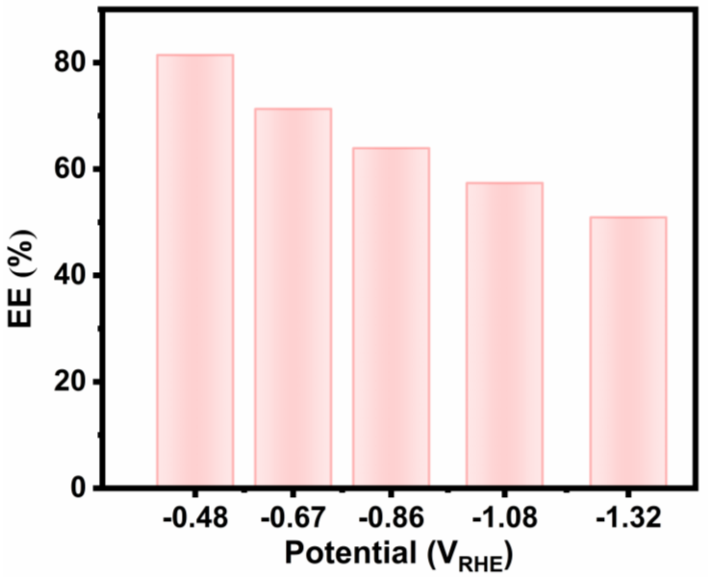

Fig. S13 EE of BiOCl nanosheet for cathodic CO_2RR in flow cell

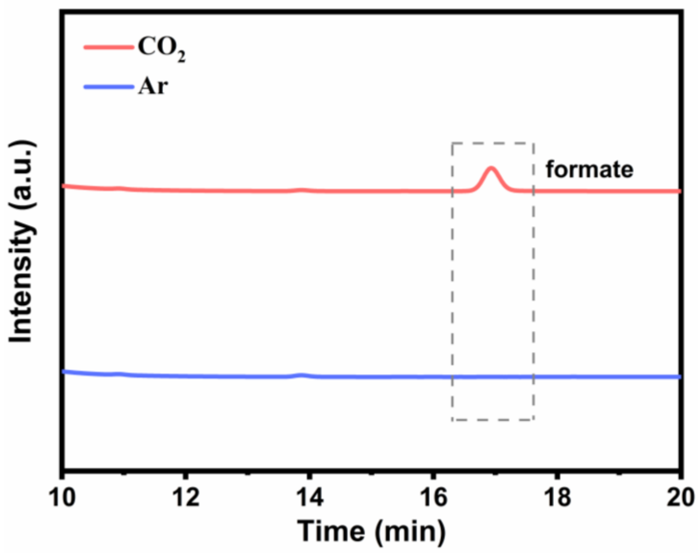

Fig. S14 HPLC spectrum of BiOCl nanosheet after electrolysis at -0.48 V_{RHE}

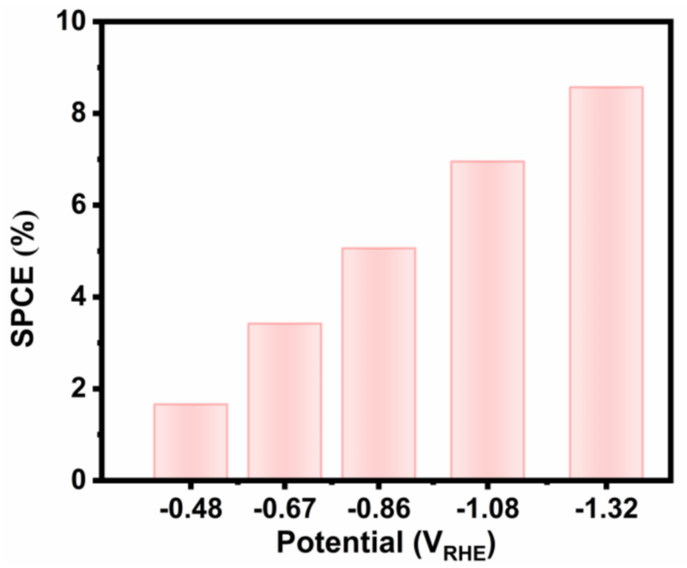

Fig. S15 SPCE of CO_2 toward formate using BiOCl nanosheet in flow cell

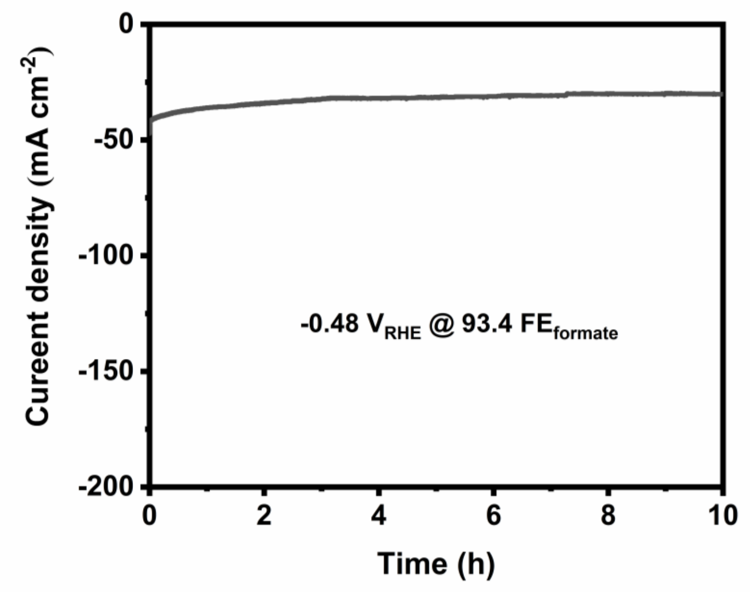

Fig. S16 Long-term stability test of BiOCl nanosheet in flow cell at -0.48 V_{RHE}

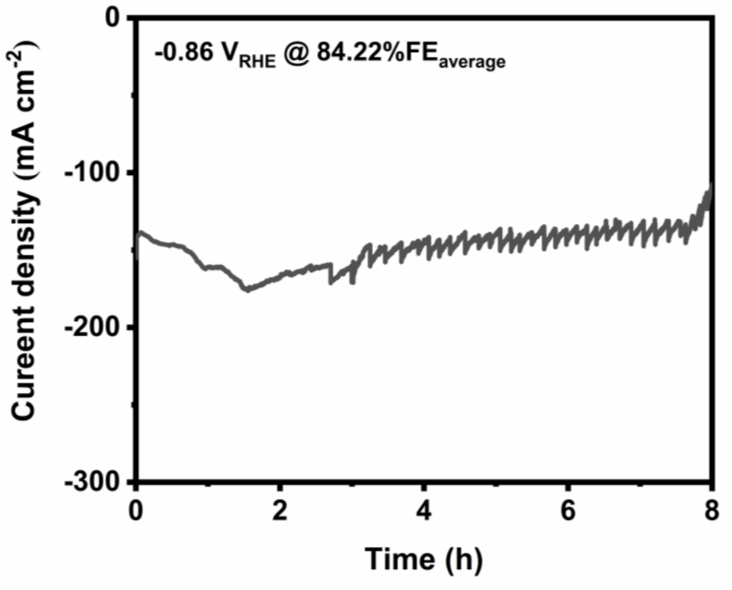

Fig. S17 Long-term stability test of BiOCl nanosheet in flow cell at -0.86 V_{RHE}

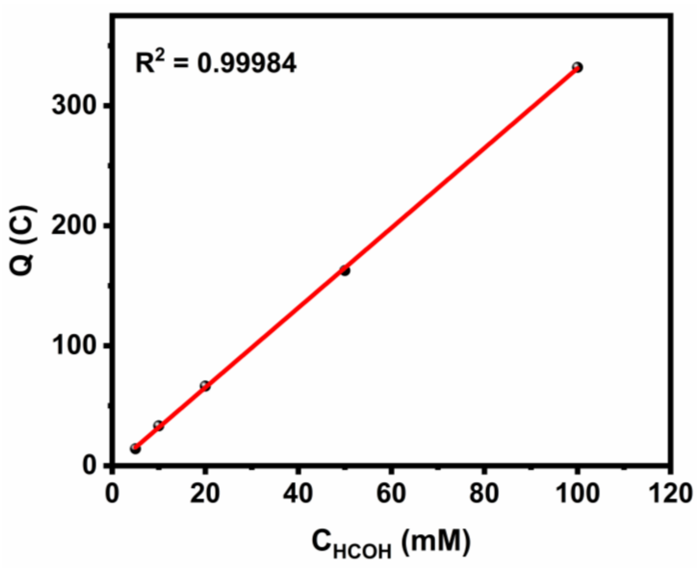

Fig. S18 Linear relationship between the concentration of formaldehyde and the charge consumed

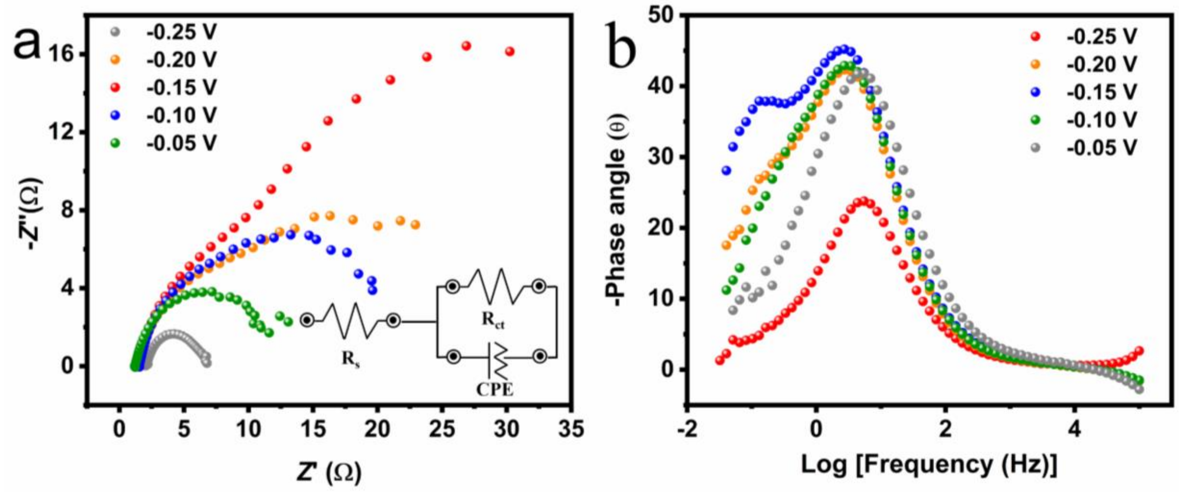

Fig. S19 (a) Nyquist plots and (**b**) Bode phase plots of Cu_2O in 1 M KOH with the addition of 100 mM formaldehyde at different potentials

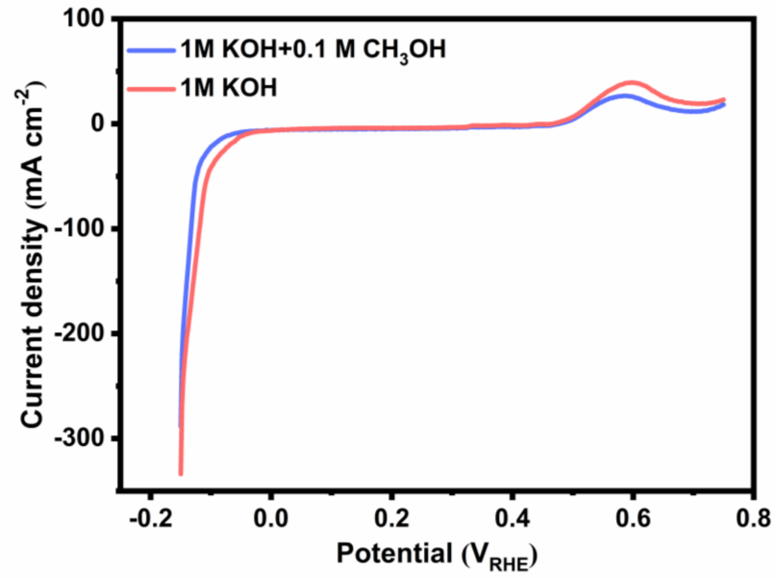

Fig. S20 LSV curves of Cu_2O in 1 M KOH electrolyte with or without CH_3OH

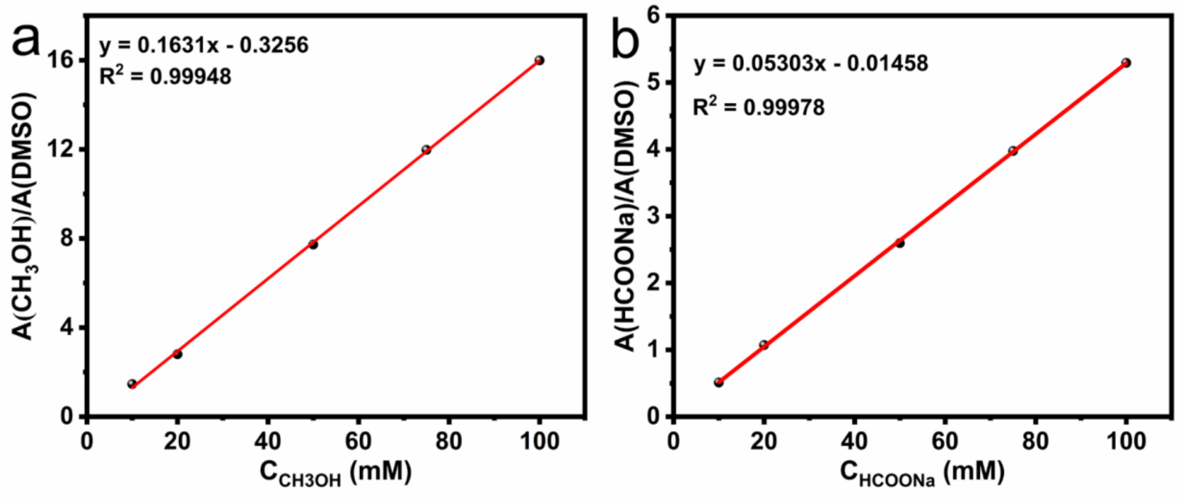

Fig. S21 (**a**) Linear relationship between the concentration of methanol and the ratio of relative area to reference sample (DMSO) area. (**b**) Linear relationship between the concentration of formate and the ratio of relative area and reference sample (DMSO) area

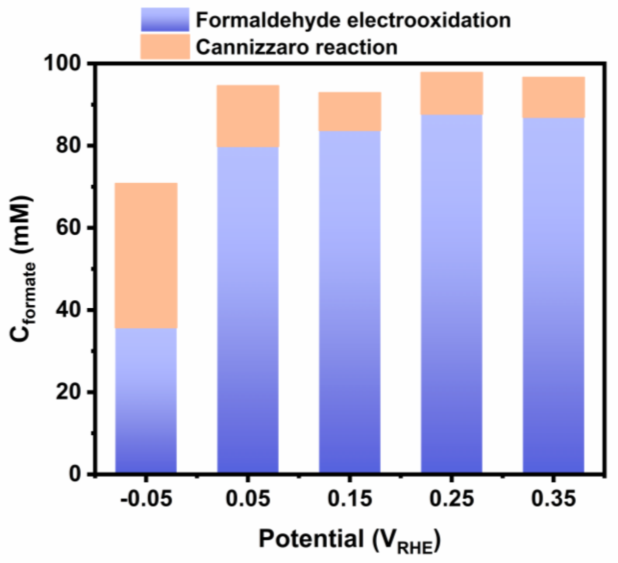

Fig. S22 Formate concentration formed by formaldehyde electrooxidation and Cannizzaro reaction

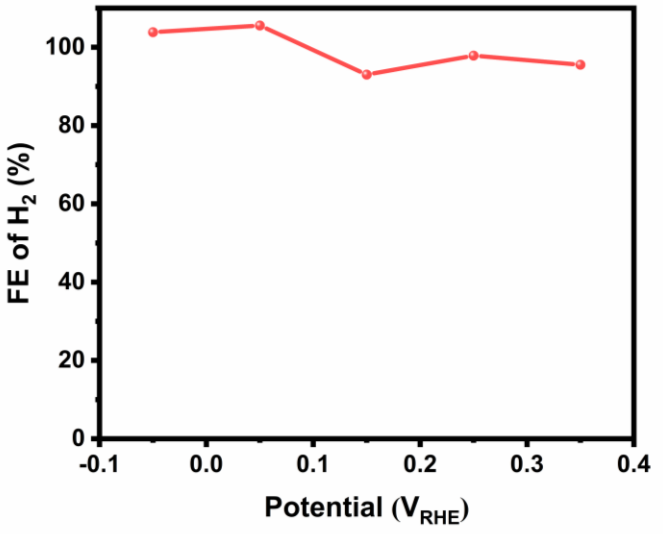

Fig. S23 H_2 FE of Cu_2O for FOR at different potentials

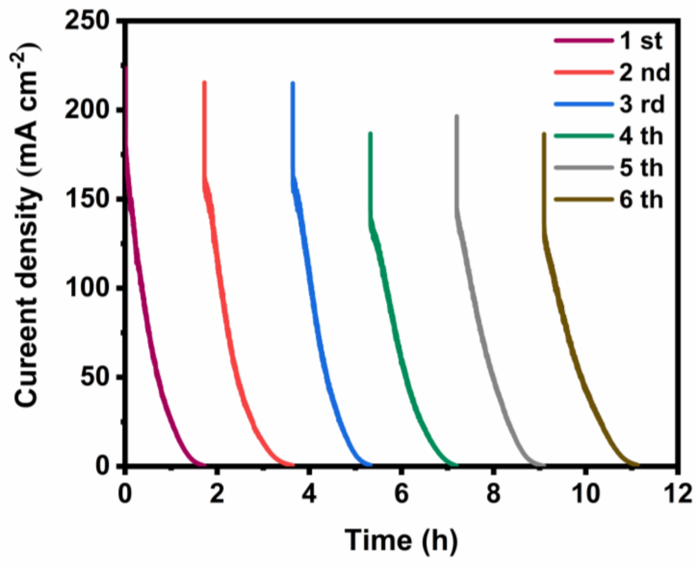

Fig. S24 *I-t* curves using Cu_2O for six successive electrolysis cycles

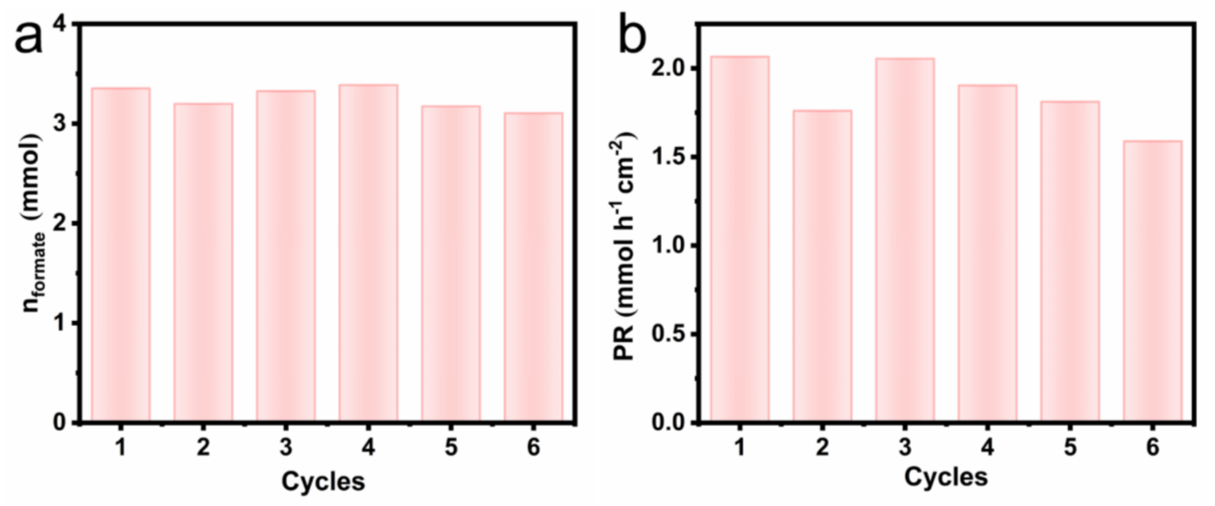

Fig. S25 (**a**) Moles and (**b**) production rate of formate using Cu_2O for six successive electrolysis cycles

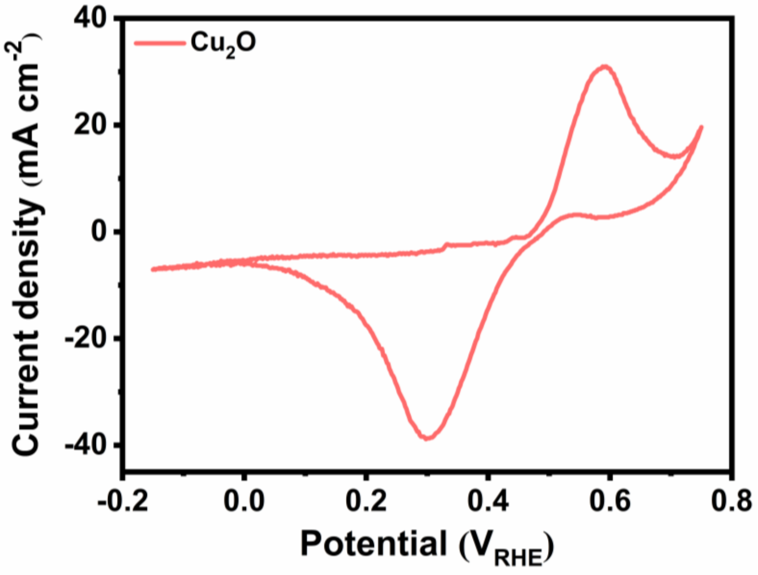

Fig. S26 CV curve of Cu_2O in 1 M KOH electrolyte

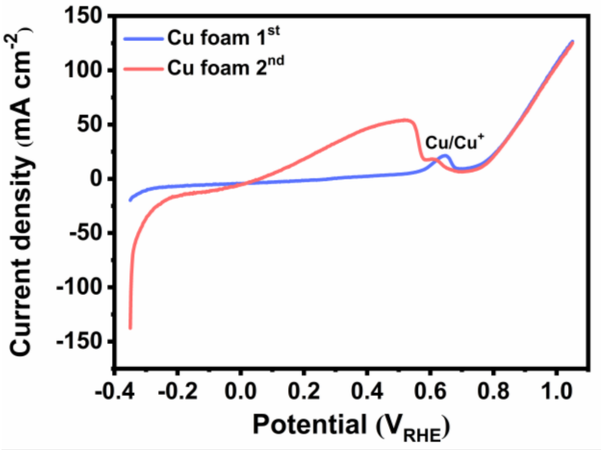

Fig. S27 LSV curves of Cu foam and Cu foam oxidation followed by reduction in 1 M KOH electrolyte with 0.1 M formaldehyde

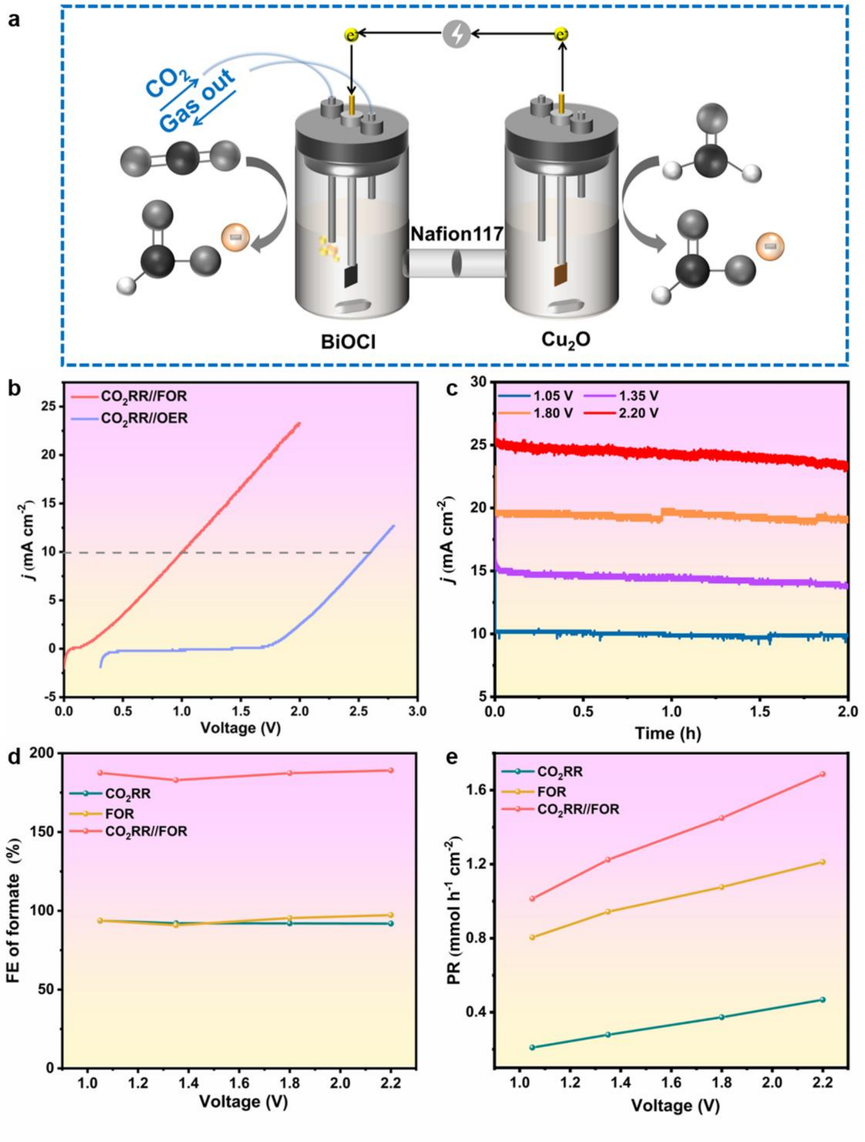

Fig. S28 Electrochemical performance of CO_2RR coupled with FOR in H-cell. (**a**) Schematic diagram for CO_2RR coupled with FOR to realize the electrosynthesis of formate. (**b**) LSV curves of CO_2RR//FOR and CO_2RR//OER full cells. (**c**) Constant potential electrolysis of CO_2RR//FOR full cell at different voltages. (**d**) Voltage-dependent formate FE, (**e**) formate production rate of anodic FOR, cathodic CO_2RR and CO_2RR//FOR full cell

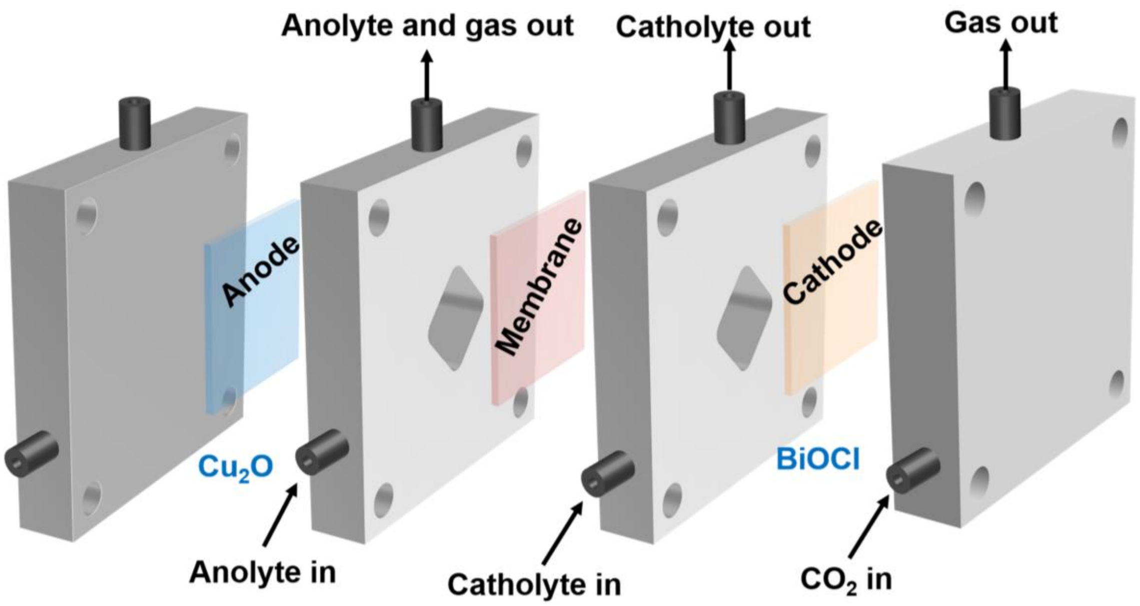

Fig. S29 Schematic diagram for CO_2RR coupled with FOR in a liquid-phase flow cell

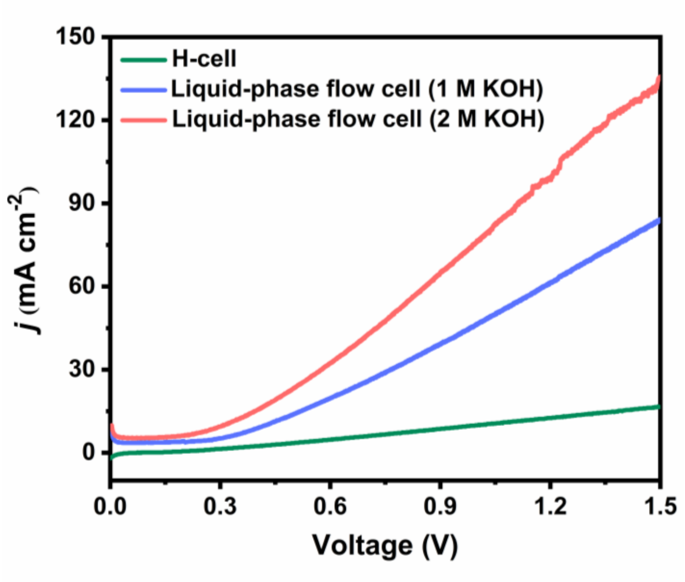

Fig. S30 LSV curves of CO_2RR//FOR full cell in H-cell and liquid-phase flow cell

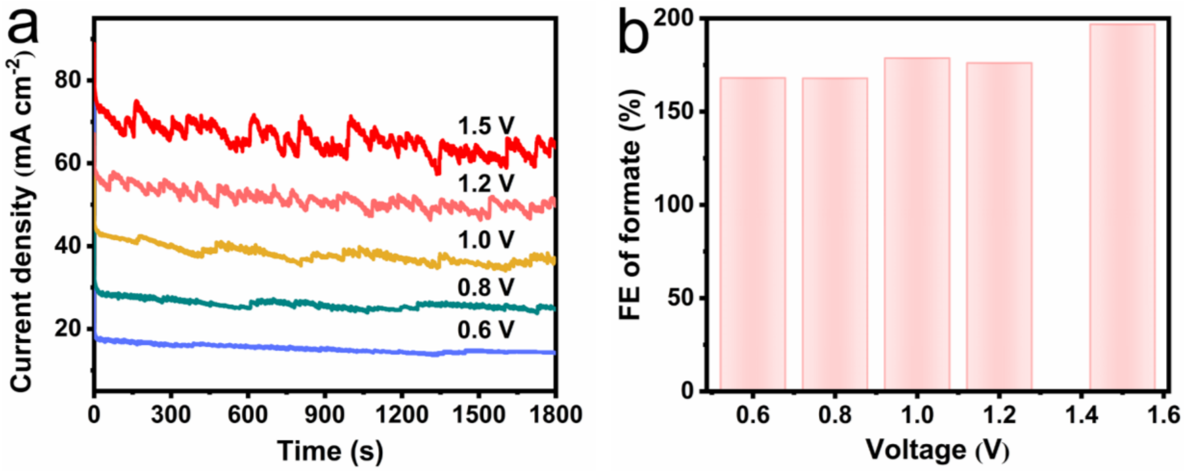

Fig. S31 (**a**) Chronoamperometry curves and (**b**) potential-dependent total formate FE in a liquid-phase flow cell with 1 M KOH electrolyte

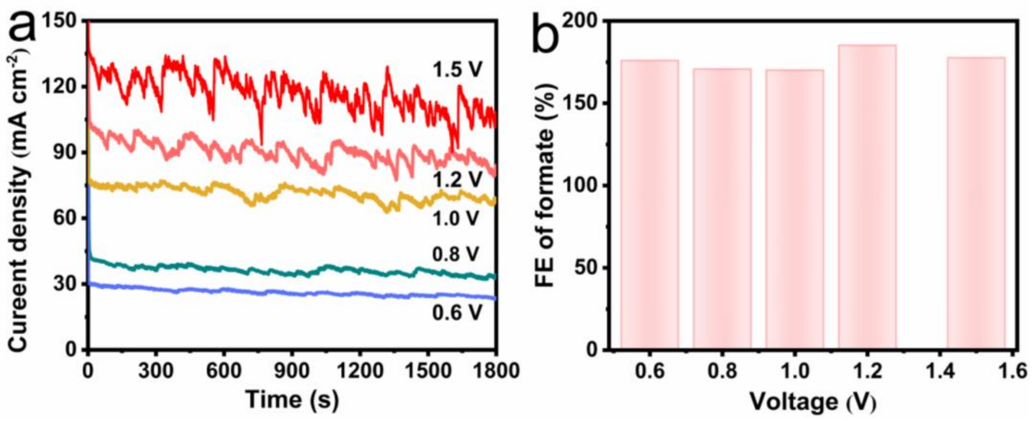

Fig. S32 (**a**) Chronoamperometry curves and (**b**) potential-dependent total formate FE in a liquid-phase flow cell with 2 M KOH electrolyte

Table S1 Gibb's free energy of formation at 25 °C and 101.325 kPa

Molecular formula	$\Delta_f G_m^0$ (kJ mol^{-1})
H_2O (l)	-237.129
O_2 (g)	0
CO_2 (g)	-394.359
HCOOH (l)	-361,35
OH^- (aq)	-157.244
HCOH (g)	-102.54

Table S2 Comparison of electrocatalytic CO_2RR to formate performance on various catalysts in H-cell from recent literature

Catalysts	Electrolyte	Potential (V_{RHE})	FE (%)	$j_{formate}$ (mA cm^{-2})	Refs.
BiOCl	0.5 M $KHCO_3$	-0.86	90.7	11.11	this work
BiOCl	0.5 M $KHCO_3$	-1.16	99.0	36.24	this work
High crystalline Bi_2O_3	0.5 M $KHCO_3$	-0.9	91	8	[S1]
Bi_2O_3@C-800	0.5 M $KHCO_3$	-0.9	92	7.5	[S2]
Bi_2O_3NSs@MCCM	0.1 M $KHCO_3$	-1.256	93.8	14	[S3]
Bi/rGO	0.1 M $KHCO_3$	-0.8	98	2~3	[S4]
Cu foam@BiNW	0.5 M $NaHCO_3$	-0.69	95	15	[S5]
$Bi_2O_2CO_3$ nanosheets	0.1 M $NaHCO_3$	-0.7	85	9.35	[S6]
Activated Bi_2Te_3 NPs/C	0.5 M $NaHCO_3$	-0.9	89.6	14	[S7]
Bi_2S_3-Bi_2O_3@rGO	0.1 M $KHCO_3$	-0.9	90.1	3.7	[S8]
Fractal-Bi_2O_3	0.1 M $KHCO_3$	-1.2	87	20.9	[S9]
Bilayer-$Bi_{12}O_{17}Cl_2$ nanosheet	0.5 M $NaHCO_3$	-0.89	93.5	11.5	[S10]
BiOBr-templated catalyst	1.0 M $KHCO_3$	-0.9	96	52.8	[S11]
Ultrathin Bi NS	0.5 M $NaHCO_3$	-0.82	95	14	[S12]
Oxide-derived Bi-Sn/CF	0.5 M $KHCO_3$	-1.14	96	43.2	[S13]
Mesoporous-SnO_2 NS	0.5 M $NaHCO_3$	-0.9	83	15	[S14]
Sn sheets/GO	0.1 M $NaHCO_3$	-1.16	89	18.8	[S15]
SnO_2 porous nanowire	0.1 M $KHCO_3$	-0.8	80	4.8	[S16]
Bi nanosheets	0.1 M $KHCO_3$	-1.1	86	16.5	[S17]
Bi dendrite	0.5 M $KHCO_3$	-0.74	89	2.7	[S18]
Bi_2S_3 derived Bi	0.5 M $NaHCO_3$	-0.75	84	4.2	[S19]
Bi nanoflakes	0.1 M $KHCO_3$	-0.6	100	2	[S20]
Bi-ene	0.5 M $KHCO_3$	-0.83	97.5	15.28	[S21]
Bi_2O_3-NGQDs	0.5 M $KHCO_3$	-0.9	98.1	18.1	[S22]

Table S3 Comparison of electrocatalytic CO_2RR to formate performance on various catalysts in flow cell from recent literature

Catalysts	Electrolyte	Potential (V_{RHE})	FE (%)	$j_{formate}$ (mA cm^{-2})	IR	Refs.
BiOCl	1 M KOH	-1.08	94.65	219.3	No	this work
MIL-68(In)-NH_2	1 M $KHCO_3$	-1.1	94	108	No	[S23]
Bi-ene	1 M KOH	-0.57	99.8	100	No	[S21]
Bi-ene	1 M KOH	-0.75	99.2	200	No	[S21]
Bi_2O_3@C-800	1 M KOH	-1.1	93	208	No	[S2]
Bi_2O_3 NTs	1 M $KHCO_3$	-0.85	95	133	90%	[S24]
Bi_2O_3 NTs	1 M KOH	-0.58	98	206	90%	[S24]
POD-Bi	1 M KOH	-0.63	93.6	187	95%	[S25]
BiOBr templated	2 M $KHCO_3$	/	90	180	90%	[S11]
Bi_2S_3	1 M KOH	-0.59	~100	205	90%	[S26]
In-Sn	0.5 M $KHCO_3$	-1.16	87.1	62.8	No	[S27]
SnO_2 nanosheet	1 M KOH	-1.13	94.2	443.7	Yes	[S28]
Sn-based GDE	0.5 M $KHCO_3$	-1.16	73.0	25.0	/	[S29]
Cu_3Sn/Cu_6Sn_5 alloy	1 M KOH	-0.98	87	128.8	/	[S30]

Table S4 The summary of the corresponding fitting date of Cu_2O electrode at different potentials

Potential (V_{RHE})	R_s (Ω)	R_{ct} (Ω)	R_{total} (Ω)
-0.25	2.064	4.65	6.714
-0.20	1.475	24.19	25.665
-0.15	1.467	46.43	47.897
-0.10	1.499	20.22	21.719
-0.05	1.202	10.80	12.002

Table S5 The summary of electrooxidation performance of Cu_2O in the complete conversion of formaldehyde under different potentials in three-electrode system

Potential V_{RHE}	C (mM)	FE (%)	Production rate (mmol h^{-1} cm^{-2})
-0.05	35.73	99.52	0.508
0.05	79.83	98.57	0.829
0.15	83.75	98.22	1.117
0.25	87.70	95.45	1.452
0.35	86.94	92.90	1.479

C is the concentration formed by formaldehyde electrooxidation.

Table S6 Comparison of electrochemical CO_2 conversion system on various catalysts from recent literature

Paired electrolysis	Anode	Cathode	Reactor	Performance	Refs.
CO_2RR//FOR	Cu_2O<br>1 M KOH+100 mM HCOH<br>FE: 93.67% ($HCOO^-$)	BiOCl<br>0.5 M $KHCO_3$<br>FE: 93.83% ($HCOO^-$)	H-cell	1.05V @ 10.6 mA cm^{-2}	this work
CO_2RR//FOR	Cu_2O<br>2 M KOH+100 mM HCOH<br>FE: 92.6% ($HCOO^-$)	BiOCl<br>2 M KOH<br>FE: 92.6% ($HCOO^-$)	Flow cell	1.2 V @ 100.2 mA cm^{-2}	this work
CO_2RR//FOR	Cu_2O<br>1 M KOH+100 mM	BiOCl<br>1 M KOH	MEA	1.0 V @ 126.9 mA cm^{-2}	this work

	HCOH<br>FE: 93.04% ($HCOO^-$)	FE: 93.04% ($HCOO^-$)			
CO_2RR//MOR	CuONS/CF<br>1 M KOH+1 M CH_3OH<br>FE: 91.3% ($HCOO^-$)	mSnO_2/CC<br>1 M $KHCO_3$<br>FE: 80.5% ($HCOO^-$)	H-cell	1.22 V@ 20 mA cm^{-2}	[S31]
CO_2RR//OER	Ir/C<br>0.5 M $KHCO_3$	BiNS<br>0.5 M $KHCO_3$<br>FE: 95% ($HCOO^-$)	H-cell	3.0 V@ 8 mA cm^{-2}	[S12]
CO_2RR//OER	RuO_2<br>1 M KOH	Bi-ene<br>0.5 M $KHCO_3$	H-cell	2.38 V @ 10 mA cm^{-2}	[S21]
CO_2RR//MOR	Ni-NF-AF<br>1 M KOH+0.5 M CH_3OH<br>FE: ~100% ($HCOO^-$)	Bi-enes<br>0.5 M $KHCO_3$<br>FE: ~100% ($HCOO^-$)	H-cell	2.13 V @ 10 mA cm^{-2}	[S32]
CO_2RR//HMF	NiO NPS<br>0.5 M $KHCO_3$+10 mM HMF<br>FE: 36% (FDCA, FFCA, DFF)	BiO_x<br>0.5 M $KHCO_3$<br>FE: 81% ($HCOO^-$)	H-cell	2.5 V @ 2 mA cm^{-2}	[S33]
CO_2RR//OER	Mesoporous-SnO_2<br>0.5 M $NaHCO_3$<br>FE: 50% (formate)	IrO_2/Ti foil<br>0.5 M $NaHCO_3$	H-cell	3 V @ 7.3 mA cm^{-2}	[S14]
CO_2RR//GOR	Pt nanoparticle<br>2 M KOH+2 M glycerol	Sn nanoparticle<br>2 M KOH<br>FE: 85.0% (HCOO-)	Flow cell	1.5 V @ 72.95 mA cm^{-2}	[S34]
CO_2RR//GOR	Pt nanoparticle<br>2 M KOH+2 M glycerol	Ag nanoparticle<br>2 M KOH<br>FE: 94.7% (CO)	Flow cell	1.5 V @ 93.42 mA cm^{-2}	[S34]
CO_2RR//UOR	Ni-WO_X<br>1 M KOH+0.33 M urea	Ag NPs<br>FE: 98% (CO)	MEA	2.16 V @ 100 mA cm^{-2}	[S35]
CO_2RR//OER	Ni-WO_X<br>1 M KOH	Ag NPs	MEA	2.53 V @ 100 mA cm^{-2}	[S35]
CO_2RR//OER	Foam nickel<br>1 M KOH	MIL-68(In)-NH_2<br>FE: 92.2% ($HCOO^-$)	MEA	2.7 V @ 258 mA cm^{-2}	[S23]

Supplementary References

[S1] P. Deng, H. Wang, R. Qi, J. Zhu, S. Chen et al., Bismuth oxides with enhanced bismuth-oxygen structure for efficient electrochemical reduction of carbon dioxide to formate. ACS Catal. **10**(1), 743-750 (2020). https://doi.org/10.1021/acscatal.9b04043

[S2] P. Deng, F. Yang, Z. Wang, S. Chen, Y. Zhou et al., Metal-organic framework-derived carbon nanorods encapsulating bismuth oxides for rapid and selective CO_2 electroreduction to formate. Angew. Chem. Int. Ed. **59**(27), 10807-10813 (2020). https://doi.org/10.1002/anie.202000657

[S3] S. Liu, X.F. Lu, J. Xiao, X. Wang, X.W. Lou, Bi_2O_3 nanosheets grown on multi-channel carbon matrix to catalyze efficient CO_2 electroreduction to HCOOH. Angew. Chem. Int. Ed. **58**(39), 13828-13833 (2019). https://doi.org/10.1002/anie.201907674

[S4] Y.X. Duan, K.H. Liu, Q. Zhang, J.M. Yan, Q. Jiang, Efficient CO_2 reduction to HCOOH with high selectivity and energy efficiency over Bi/rGO catalyst. Small Methods **4**(5), 1900846 (2020). https://doi.org/10.1002/smtd.201900846

[S5] X. Zhang, X. Sun, S.X. Guo, A.M. Bond, J. Zhang, Formation of lattice-dislocated bismuth nanowires on copper foam for enhanced electrocatalytic CO_2 reduction at low overpotential. Energy Environ. Sci. **12**(4), 1334-1340 (2019). https://doi.org/10.1039/c9ee00018f

[S6] Y. Zhang, X. Zhang, Y. Ling, F. Li, A.M. Bond et al., Controllable synthesis of few-layer bismuth subcarbonate by electrochemical exfoliation for enhanced CO_2 reduction performance. Angew. Chem. Int. Ed. **57**(40), 13283-13287 (2018). https://doi.org/10.1002/anie.201807466

[S7] N. Zhang, F. Zheng, B. Huang, Y. Ji, Q. Shao et al., Exploring Bi_2Te_3 nanoplates as versatile catalysts for electrochemical reduction of small molecules. Adv. Mater. **32**(22), 1906477 (2020). https://doi.org/10.1002/adma.201906477

[S8] X. Yang, P. Deng, D. Liu, S. Zhao, D. Li et al., Partial sulfuration-induced defect and interface tailoring on bismuth oxide for promoting electrocatalytic CO_2 reduction. J. Mater. Chem. A **8**(5), 2472-2480 (2020). https://doi.org/10.1039/c9ta11363k

[S9] T. Tran-Phu, R. Daiyan, Z. Fusco, Z. Ma, R. Amal et al., Nanostructured β-Bi_2O_3 fractals on carbon fibers for highly selective CO_2 electroreduction to formate. Adv. Funct. Mater. **30**(3), 1906478 (2020). https://doi.org/10.1002/adfm.201906478

[S10] J. Zhu, J. Fan, T. Cheng, M. Cao, Z. Sun et al., Bilayer nanosheets of unusual stoichiometric bismuth oxychloride for potassium ion storage and CO_2 reduction. Nano Energy **75**, 104939 (2020). https://doi.org/10.1016/j.nanoen.2020.104939

[S11] F.P.G.D. Arquer, O.S. Bushuyev, P.D. Luna, C.T. Dinh, A. Seifitokaldani et al., 2D metal oxyhalide-derived catalysts for efficient CO_2 electroreduction. Adv. Mater. **30**(38), 1802858 (2018). https://doi.org/10.1002/adma.201802858

[S12] N. Han, Y. Wang, H. Yang, J. Deng, J. Wu et al., Ultrathin bismuth nanosheets from in situ topotactic transformation for selective electrocatalytic CO_2 reduction to formate. Nat. Commun. **9**, 1320 (2018). https://doi.org/10.1038/s41467-018-03712-z

[S13] G. Wen, D.U. Lee, B. Ren, F.M. Hassan, G. Jiang et al., Orbital interactions in Bi-Sn bimetallic electrocatalysts for highly selective electrochemical CO_2 reduction toward formate production. Adv. Energy Mater. **8**(31), 1802427 (2018). https://doi.org/10.1002/aenm.201802427

[S14] N. Han, Y. Wang, J. Deng, J. Zhou, Y. Wu et al., Self-templated synthesis of hierarchical mesoporous SnO_2 nanosheets for selective CO_2 reduction. J. Mater. Chem. A **7**(3), 1267-1272 (2019). https://doi.org/10.1039/c8ta10959a

[S15] F. Lei, W. Liu, Y. Sun, J. Xu, K. Liu et al., Metallic tin quantum sheets confined in graphene toward high-efficiency carbon dioxide electroreduction. Nat. Commun. **7**, 12697 (2016). https://doi.org/10.1038/ncomms12697

[S16] B. Kumar, V. Atla, J.P. Brian, S. Kumari, T.Q. Nguyen et al., Reduced SnO_2 porous nanowires with a high density of grain boundaries as catalysts for efficient electrochemical CO_2-into-HCOOH Conversion. Angew. Chem. Int. Ed. **56**(13), 3645-3649 (2017). https://doi.org/10.1002/anie.201612194

[S17] W. Zhang, Y. Hu, L. Ma, G. Zhu, P. Zhao et al., Liquid-phase exfoliated ultrathin Bi nanosheets: uncovering the origins of enhanced electrocatalytic CO_2 reduction on two-dimensional metal nanostructure. Nano Energy **53**, 808-816 (2018). https://doi.org/10.1016/j.nanoen.2018.09.053

[S18] J.H. Koh, D.H. Won, T. Eom, N.K. Kim, K.D. Jung et al., Facile CO_2 electro-reduction to formate via oxygen bidentate intermediate stabilized by high-index planes of Bi

dendrite catalyst. ACS Catal. **7**(8), 5071-5077 (2017). https://doi.org/10.1021/acscatal.7b00707

[S19] Y. Zhang, F. Li, X. Zhang, T. Williams, C.D. Easton et al., Electrochemical reduction of CO_2 on defect-rich Bi derived from Bi_2S_3 with enhanced formate selectivity. J. Mater. Chem. A **6**(11), 4714-4720 (2018). https://doi.org/10.1039/c8ta00023a

[S20] S. Kim, W.J. Dong, S. Gim, W. Sohn, J.Y. Park et al., Shape-controlled bismuth nanoflakes as highly selective catalysts for electrochemical carbon dioxide reduction to formate. Nano Energy **39**, 44-52 (2017). https://doi.org/10.1016/j.nanoen.2017.05.065

[S21] C. Cao, D.D. Ma, J.F. Gu, X. Xie, G. Zeng et al., Metal-organic layers leading to atomically thin bismuthene for efficient carbon dioxide electroreduction to liquid fuel. Angew. Chem. Int. Ed. **59**(35), 15014-15020 (2020). https://doi.org/10.1002/anie.202005577

[S22] Z. Chen, K. Mou, X. Wang, L. Liu, Nitrogen-doped graphene quantum dots enhance the activity of Bi_2O_3 nanosheets for electrochemical reduction of CO_2 in a wide negative potential region. Angew. Chem. Int. Ed. **57**(39), 12790-12794 (2018). https://doi.org/10.1002/anie.201807643

[S23] Z. Wang, Y. Zhou, C. Xia, W. Guo, B. You et al., Efficient electroconversion of carbon dioxide to formate by a reconstructed amino-functionalized indium-organic framework electrocatalyst. Angew. Chem. Int. Ed. **60**(35), 19107-19112 (2021). https://doi.org/10.1002/anie.202107523

[S24] Q. Gong, P. Ding, M. Xu, X. Zhu, M. Wang et al., Structural defects on converted bismuth oxide nanotubes enable highly active electrocatalysis of carbon dioxide reduction. Nat. Commun. **10**, 2807 (2019). https://doi.org/10.1038/s41467-019-10819-4

[S25] S. He, F. Ni, Y. Ji, L. Wang, Y. Wen et al., The p-orbital delocalization of main-group metals to boost CO_2 electroreduction. Angew. Chem. Int. Ed. **57**(49), 16114-16119 (2018). https://doi.org/10.1002/anie.201810538

[S26] P. Ding, J. Zhang, N. Han, Y. Zhou, L. Jia et al., Simultaneous power generation and CO_2 valorization by aqueous Al-CO_2 batteries using nanostructured Bi_2S_3 as the cathode electrocatalyst. J. Mater. Chem. A **8**(25), 12385-12390 (2020). https://doi.org/10.1039/d0ta03761c

[S27] Q. Wang, Y. Wu, C. Zhu, R. Xiong, Y. Deng et al., Sn nanoparticles deposited onto a gas diffusion layer via impregnation-electroreduction for enhanced CO_2 electroreduction to formate. Electrochim. Acta **369**(10), 137662 (2021). https://doi.org/10.1016/j.electacta.2020.137662

[S28] J. Li, J. Jiao, H. Zhang, P. Zhu, H. Ma et al., Two-dimensional SnO_2 nanosheets for efficient carbon dioxide electroreduction to formate. ACS Sustainable Chem. Eng. **8**(12), 4975-4982 (2020). https://doi.org/10.1021/acssuschemeng.0c01070

[S29] Q. Wang, X. Wang, C. Wu, Y. Cheng, Q. Sun et al., Electrodeposition of tin on Nafion-bonded carbon black as an active catalyst layer for efficient electroreduction of CO_2 to formic acid. Sci. Rep. **7**, 13711 (2017). https://doi.org/10.1038/s41598-017-14233-y

[S30] J. Wang, J. Zou, X. Hu, S. Ning, X. Wang et al., Heterostructured intermetallic CuSn catalysts: high performance towards the electrochemical reduction of CO_2 to formate. J. Mater. Chem. A **7**(48), 27514-27521 (2019). https://doi.org/10.1039/c9ta11140a

[S31] X. Wei, Y. Li, L. Chen, J. Shi, Formic acid electro-synthesis by concurrent cathodic CO_2 reduction and anodic CH_3OH oxidation. Angew. Chem. Int. Ed. **60**(6), 3148-3155 (2021). https://doi.org/10.1002/anie.202012066

[S32] C. Cao, D.D. Ma, J. Jia, Q. Xu, X.T. Wu et al., Divergent paths, same goal: a pair-electrosynthesis tactic for cost-efficient and exclusive formate production by metal-organic-framework-derived 2D electrocatalysts. Adv. Mater. **33**(25), 2008631 (2021). https://doi.org/10.1002/adma.202008631

[S33] S. Choi, M. Balamurugan, K.G. Lee, K.H. Cho, S. Park et al., Mechanistic investigation of biomass oxidation using nickel oxide nanoparticles in a CO_2-saturated electrolyte for paired electrolysis. J. Phys. Chem. Lett. **11**(8), 2941-2948 (2020). https://doi.org/10.1021/acs.jpclett.0c00425

[S34] S. Verma, S. Lu, P.J.A. Kenis, Co-electrolysis of CO_2 and glycerol as a pathway to carbon chemicals with improved technoeconomics due to low electricity consumption. Nat. Energy **4**(6), 466-474 (2019). https://doi.org/10.1038/s41560-019-0374-6

[S35] L. Wang, Y. Zhu, Y. Wen, S. Li, C. Cui et al., Regulating the local charge distribution of Ni active sites for the urea oxidation reaction. Angew. Chem. Int. Ed. **60**(19), 10577-10582 (2021). https://doi.org/10.1002/anie.202100610
